# Long noncoding RNA BCYRN1 promotes cardioprotection by enhancing human and murine regulatory T cell dynamics

**DOI:** 10.1172/JCI179262

**Published:** 2025-03-25

**Authors:** Ke Liao, Jiayi Yu, Akbarshakh Akhmerov, Zahra Mohammadigoldar, Liang Li, Weixin Liu, Natasha Anders, Ahmed G.E. Ibrahim, Eduardo Marbán

**Affiliations:** Smidt Heart Institute, Cedars-Sinai Medical Center, Los Angeles, California, USA.

**Keywords:** Cardiology, Therapeutics, Autophagy, Gene therapy, T cells

## Abstract

Regulatory T cells (Tregs) modulate immune responses and attenuate inflammation. Extracellular vesicles from human cardiosphere-derived cells (CDC-EVs) enhance Treg proliferation and IL-10 production, but the mechanisms remain unclear. Here, we focused on BCYRN1, a long noncoding RNA (lncRNA) highly abundant in CDC-EVs, and its role in Treg function. BCYRN1 acts as a “microRNA sponge,” inhibiting miR-138, miR-150, and miR-98. Suppression of these miRs leads to increased Treg proliferation via ATG7-dependent autophagy, CCR6-dependent Treg migration, and enhanced Treg IL-10 production. In a mouse model of myocardial infarction, CDC-EVs, particularly those overexpressing BCYRN1, were cardioprotective, reducing infarct size and troponin I levels even when administered after reperfusion. Underlying the cardioprotection, we verified that CDC-EVs overexpressing BCYRN1 increased cardiac Treg infiltration, proliferation, and IL-10 production in vivo. These salutary effects were negated when BCYRN1 levels were reduced in CDC-EVs or when Tregs were depleted systemically. Thus, we have identified BCYRN1 as a booster of Treg number and bioactivity, rationalizing its cardioprotective efficacy. While we studied BCYRN1 overexpression in the context of ischemic injury here, the same approach merits testing in other disease processes (e.g., autoimmunity or transplant rejection) where increased Treg activity is a recognized therapeutic goal.

## Abstract 2

RNA-based therapeutics: BCYRN1, a long noncoding RNA in CDC-EVs, boosts Treg functions, offering potential treatments for cardiac and autoimmune diseases

## Graphical abstract

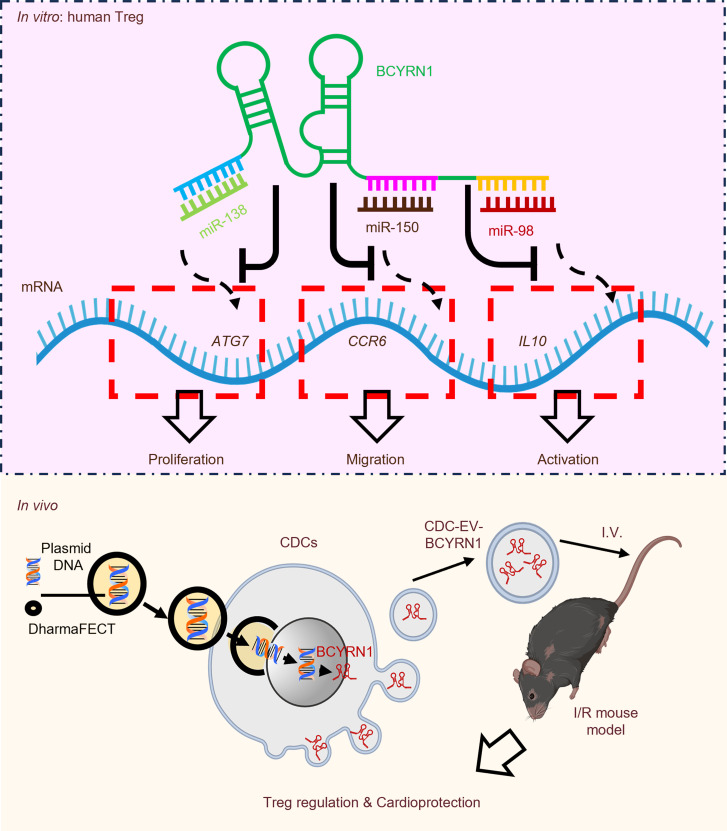

## Introduction

Myocardial infarction (MI) triggers a robust innate immune response that releases proinflammatory cytokines and recruits pathologically activated neutrophils, macrophages, dendritic cells, and T cells to the myocardium (1–3). In response, the heart dilates and weakens after MI, predisposing to heart failure. Regulatory T cells (Tregs), especially CD4^+^Foxp3^+^ Tregs with immunosuppressive properties, offset excessive myocardial inflammation (4) by releasing inhibitory cytokines (e.g., IL-10) and growth factors (e.g., TGFB) or via contact-dependent effects (5, 6). Autologous Treg transplantation has been attempted for a variety of chronic diseases, but clinical trials have been challenged by difficulties in Treg isolation and ex vivo expansion (7–9). Given these limitations, which introduce delays of several weeks from harvesting to clinical readiness, autologous Treg infusion is not a realistic therapeutic principle for acute disease such as MI. Approaches to recruit Tregs in vivo are clearly desirable.

Cardiosphere-derived cells (CDCs) are stromal/progenitor cells, now in advanced clinical testing for Duchenne muscular dystrophy (clinicaltrials.gov NCT05126758) (10), with antiinflammatory and immunomodulatory properties mediated by secreted extracellular vesicles (EVs) (11). Such EVs from human CDCs (CDC-EVs) increase proliferation of Tregs and augment their production of IL-10, which, in turn, attenuates cardiac inflammation in rodent models of myocarditis (12). However, the mechanism underlying CDC-EV–mediated recruitment and activation of Tregs is unknown. CDC-EVs transmit a diverse cargo of noncoding RNAs and other bioactive molecules into adjacent cells to regulate their biology, highlighting a noncanonical means of cellular crosstalk (13–18). Short noncoding RNAs mediate many of the salient benefits of CDC-EVs: as two examples, microRNA-146a (miR-146a) within CDC-EVs promotes cardiac regeneration in vivo (19) and EV-YF1, a Y RNA species, is cardioprotective and antihypertrophic (20–22). However, long noncoding RNAs (lncRNAs), i.e., those ≥200 nucleotides in length, have remained unexplored as potential mediators of CDC-EV bioactivity. Here, we tested the hypothesis that a plentiful lncRNA underlies CDC-EVs’ ability to upregulate Treg number and activity in the acute setting.

## Results

### CDC-EVs induce Treg proliferation, migration, and upregulation of IL-10.

**Figure 1:**
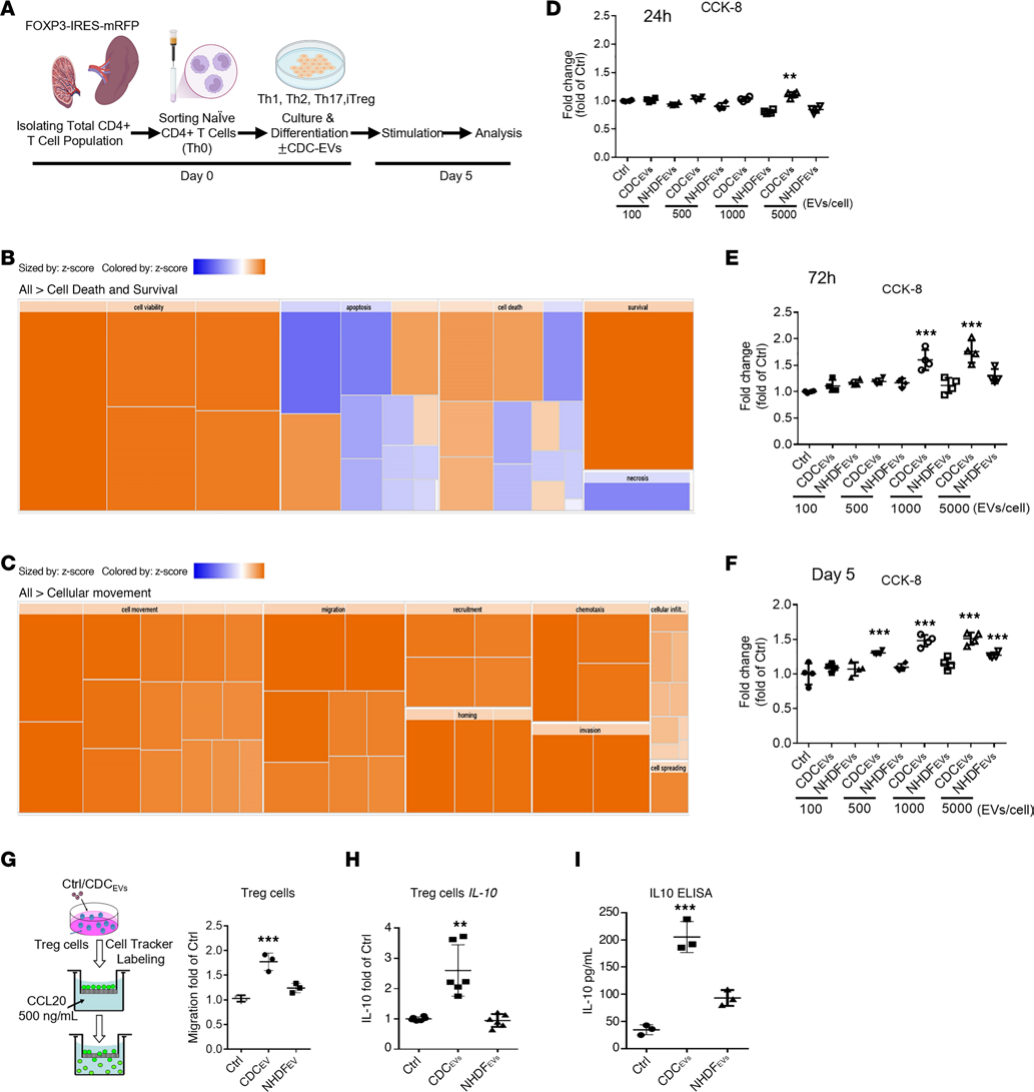
CDC-EVs induce human iTreg proliferation, migration, and induction of IL-10. (**A**) Experimental outline for in vitro Treg global transcriptomic analysis: total CD4^+^ T cell population isolated from FOXP3 reporter mice, followed by differentiating the isolated naive CD4^+^ T cells into induced Tregs (iTregs). The iTregs were exposed either to vehicle or human CDC-EVs for 5 days and subsequently assessed for transcriptomic changes. (**B** and **C**) Functional heatmap depicting enrichment in cell survival (**B**) and cellular movement (**C**) categories in the upregulated transcripts in CDC-EVs versus vehicle based on IPA analysis. (**D**–**F**) Human iTregs were exposed either to CDC-EVs or fibroblast EVs (NHDF-EVs) at indicated concentrations (0–5,000 EVs/cell) for 24 hours (**D**), 72 hours (**E**), and 5 days (**F**). (**G**) The migration of human iTregs following exposure to CDC-EVs or NHDF-EVs, toward 500 ng/mL recombinant CCL20, was assessed using a 24-well Transwell plate. (**H**) Real-time PCR analysis of IL-10 mRNA expression in human iTregs exposed to either CDC-EVs or NHDF-EVs for 72 hours. (**I**) Protein levels of IL-10 were assayed by ELISA in supernatants of human iTregs exposed to either CDC-EVs or NHDF-EVs for 72 hours. One-way ANOVA followed by Bonferroni’s post hoc test was used to determine statistical significance. All data are presented as mean ± SEM of 3 or 4 individual experiments (biological replicates). *P < 0.05, **P < 0.01, ***P < 0.001 versus control group.

In a prior work (12), we isolated naive mouse Cd4^+^ T cells from mice and differentiated them into conventional effector T cells (Th1, Th2, Th17) or induced Tregs (iTregs) (Figure 1A). After 5 days of culture, exposure of cells to CDC-EVs resulted in distinct, dose-dependent responses among the different types of CD4^+^ T cells: no change in Th1 and Th2 proliferation; decreased proliferation of Th17 cells; and increased proliferation of iTregs (dosing at 1,000 EVs/cell elicited a strong proliferative response). Using RNA-Seq, we also discovered that CDC-EVs could augment IL-10 production by Tregs. New ingenuity pathway analysis (IPA) of the RNA-Seq data (12) (Figure 1, B and C) from iTregs revealed several significantly upregulated categories (based on *z* score), which are represented by progressively brighter orange-to-red colors as the upregulation increases. Conversely, decreased expression is depicted in blue. Heatmaps showed that CDC-EVs increased the expression of genes promoting cell survival and viability but decreased expression of genes related to apoptosis and necrosis (Figure 1B). Additionally, CDC-EVs strongly induced genes promoting cell movement and migration (Figure 1C). Motivated by these findings, we performed new experiments to test whether CDC-EVs induce cell proliferation, migration, and IL-10 production in human iTregs exposed to different doses (0–5,000 EVs/cell) of CDC-EVs for various durations (0–5 days). Normal human dermal fibroblast EVs (NHDF-EVs) served as a negative control; these EVs are similar in size distribution but functionally inert in the relevant assays (19). Exposure of human iTregs to CDC-EVs resulted in a dose-dependent increase in cell number over time (Figure 1, D–F). Trans-well experiments confirmed that CDC-EVs induced human iTreg migration (Figure 1G), *IL-10* gene expression (Figure 1H), and IL-10 protein secretion (Figure 1I).

### CDC-EV–mediated expression of BCYRN1 in human iTregs.

**Figure 2:**
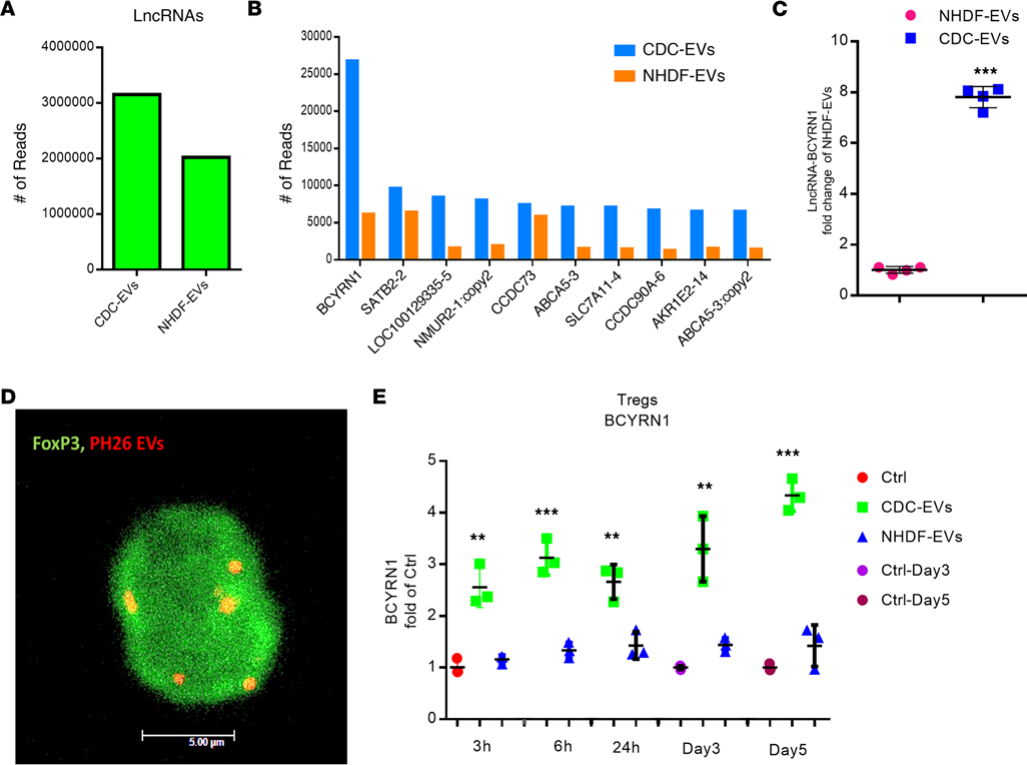
CDC-EVs mediate increased expression of BCYRN1 in Tregs. (**A**) RNA-Seq of CDC-EVs shows lncRNA to be plentiful compared with NHDF-EVs. (**B**) List of top 10 most plentiful lncRNAs, among which BCYRN1 is the highest enriched in CDC-EVs (CDC-EVs [blue bars] and NHDF-EVs [orange bars]). (**C**) BCYRN1 is expressed more in CDC-EVs than in NHDF-EVs by qPCR. (**D**) Confocal microscopy shows uptake of PKH26-labeled CDC-EVs by human iTregs. Scale bar: 5 μm. (**E**) Assessment of BCYRN1 expression by qPCR in human iTregs exposed to CDC-EVs or NHDF-EVs for indicated times. One-way ANOVA followed by Bonferroni’s post hoc test was used to assess statistical significance. All data are presented as mean ± SEM of 3 or 4 individual experiments (biological replicates). *P < 0.05, **P < 0.01, ***P < 0.001 versus control (Ctrl) group.

To dissect the mechanism whereby CDC-EVs enhance human iTregs, we performed RNA-Seq. CDC-EVs contain many (>10,000) molecularly distinct noncoding RNA (ncRNA) entities with known or plausible bioactivity (20). We look beyond miRs, which are the “usual suspects” in EV cargo studies, because they are well studied and relatively scarce (20). RNA-Seq revealed that lncRNAs were abundant in CDC-EVs (~50% more plentiful than in inert NHDF-EVs; Figure 2A). RNA-Seq library data (Figure 2B) quantifying lncRNAs from CDC-EVs and NHDF-EVs revealed one particular species — BCYRN1 — to be the most plentiful lncRNA in CDC-EVs, a finding confirmed by qPCR (Figure 2C) using *GAPDH* as the internal control to normalize the lncRNA expression levels (23, 24). Next, we investigated whether BCYRN1 within CDC-EVs could be transferred from CDCs to human iTregs, leading, in turn, to enhanced levels of BCYRN1 in recipient cells. Human iTregs (labeled for Treg marker Foxp3) exposed to CDC-EVs and labeled with PKH26 revealed cytoplasmic puncta consistent with EV internalization (Figure 2D). Exposure to unlabeled CDC-EVs resulted in time-dependent increases in BCYRN1 in human iTregs (Figure 2E). Cellular levels of BCYRN1 were enhanced up to 4-fold; the effect was selective, as exposure to NHDF-EVs caused no significant changes in BCYRN1 levels. Thus, BCYRN1 is highly enriched in CDC-EVs; uptake of these EVs increases BCYRN1 levels in human iTregs.

### CDC-EV–mediated human iTreg proliferation, migration, and induction of IL-10 involve BCYRN1.

**Figure 3:**
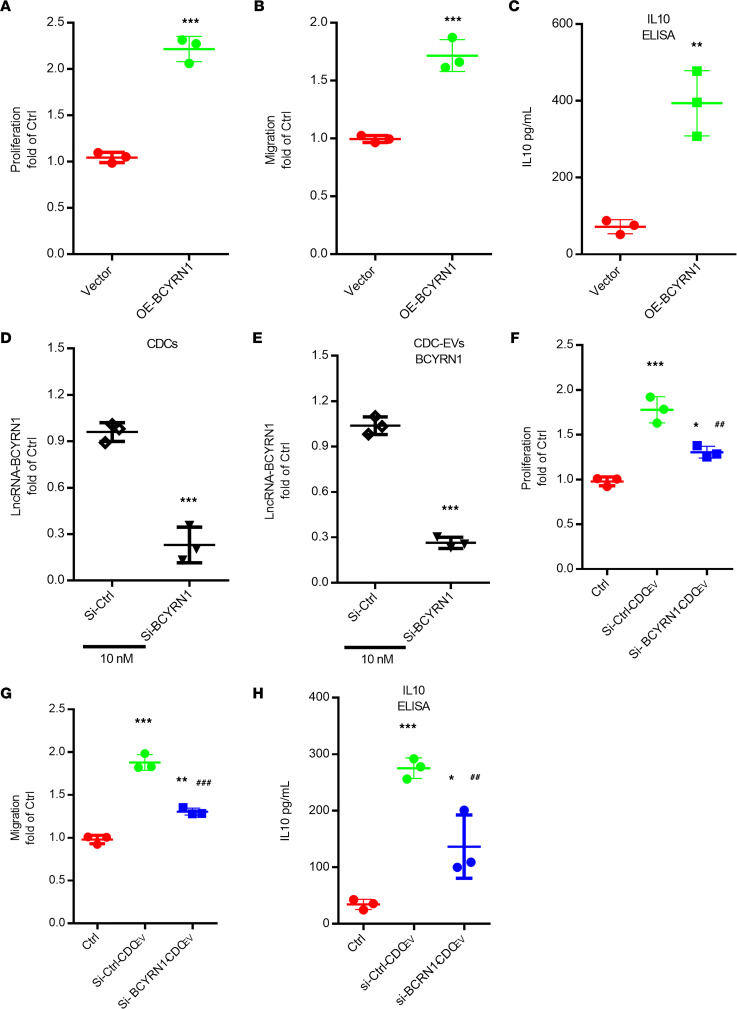
CDC-EV–mediated Treg proliferation, migration, and induction of IL-10 involves BCYRN1. (**A**–**C**) Effects of overexpression (OE) of BCYRN1 in human iTregs on proliferation (**A**), migration (**B**), and IL-10 production (**C**). (**D** and **E**) Transfection of CDCs with siRNA-BCYRN1 results in knockdown of BCYRN1 in both CDCs and CDC-EVs. (**F**–**H**) Exposure of human iTregs to CDC-EVs with BCYRN1 knockdown followed by assessments of proliferation (**F**), migration (**G**), and IL-10 production (**H**). One-way ANOVA followed by Bonferroni’s post hoc test was used to assess statistical significance. All data are presented as mean ± SEM of 3 or 4 individual experiments (biological replicates). *P < 0.05, **P < 0.01, ***P < 0.001 versus control group. ^##^*P* < 0.01 versus si-Ctrl-CDC-EVs group.

We next sought to determine whether BCYRN1 was responsible for CDC-EV–induced upregulation of human iTregs by directly transfecting human iTregs with either empty lentiviral vector or vector expressing BCYRN1. Overexpression of BCYRN1 increased human iTreg proliferation (Figure 3A), migration (Figure 3B), and IL-10 production (Figure 3C). To further probe BCYRN1’s role in human iTreg modulation, we used siRNA to suppress BCYRN1. Transfection of CDCs with siRNA against BCYRN1 (si-BCYRN1) for 48 hours, followed by EV isolation, revealed decreased expression of BCYRN1 both in CDCs (Figure 3D) and CDC-EVs (Figure 3E) relative to siRNA-control (si-ctrl) group. In terms of Treg proliferation, migration, or IL-10 production, EVs with siRNA-suppressed BCYRN1 elicited much weaker responses than CDC-EVs with normal BCYRN1 levels (i.e., those from CDCs exposed only to si-ctrl; Figure 3, F–H). Thus, BCYRN1 underlies the ability of CDC-EVs to enhance human iTreg number and bioactivity.

### CDC-EV BCYRN1 induces ATG7-dependent autophagy by sponging miR-138.

**Figure 4:**
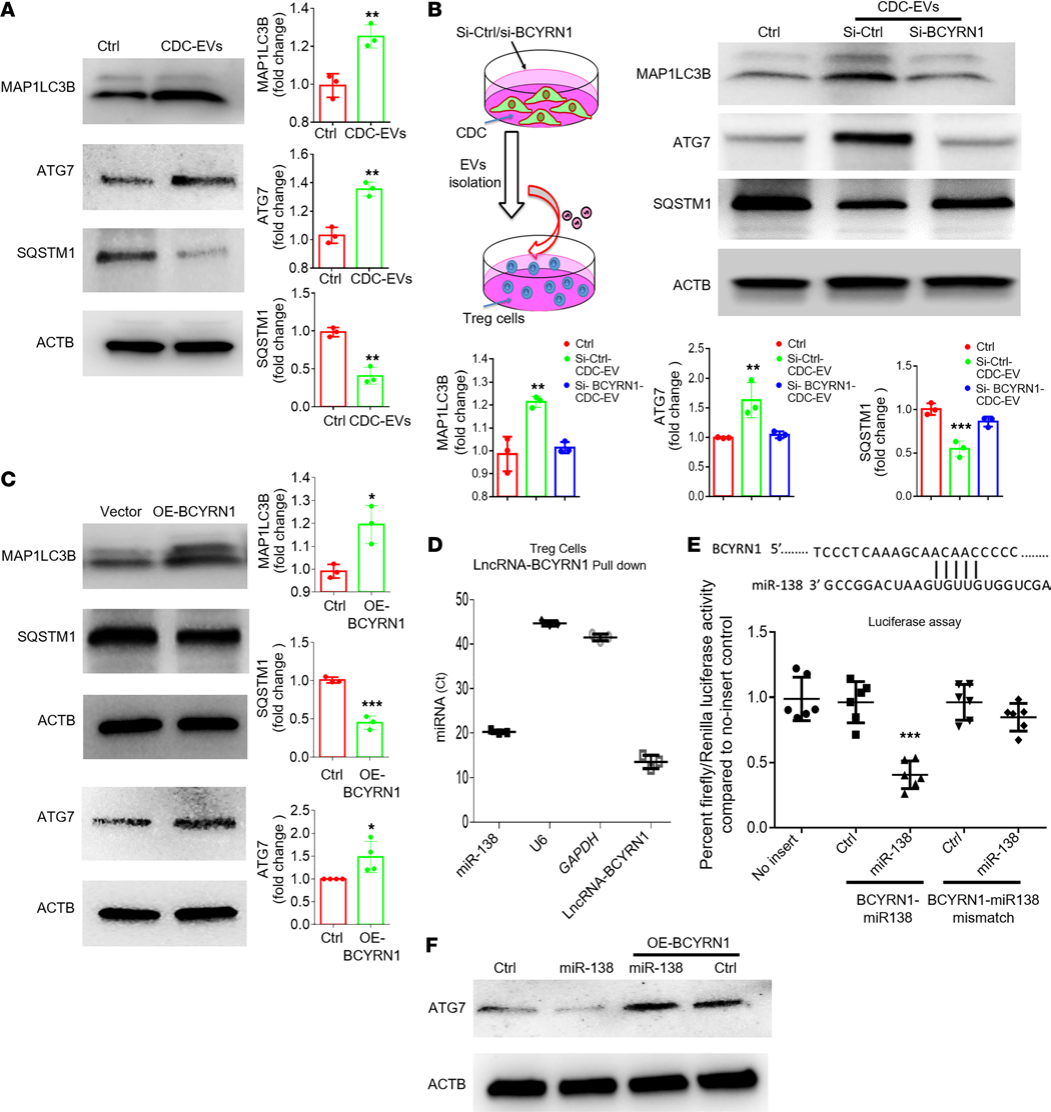
CDC-EV BCYRN1 induces autophagy by competitively binding miR-138 to regulate ATG7 expression. (**A**) Human iTregs were exposed to CDC-EVs or not treated (ctrl). The expression of autophagy markers (MAP1LC3B, ATG7, and SQSTM1) was assessed by Western blot (WB). (**B**) Human iTregs were exposed to ctrl-CDC-EVs or si-BCYRN1 CDC-EVs (EV with BCYRN1 knockdown), or were not treated (ctrl). Expression of autophagy markers (MAP1LC3B, ATG7, and SQSTM1) was assessed by WB. (**C**) Human iTregs were transfected with vector or OE-BCYRN1 lenti-vector, followed by assessment of autophagy markers by WB. (**D**) Biotin-labeled BCYRN1 probe was used to pull down BCYRN1-associated RNAs, followed by assessment of miR-138, negative control (*U6* and *GAPDH*), and positive control (BCYRN1) expression by qPCR. (**E**) The top panel shows putative lncRNA BCYRN1 binding sites in miR-138. The bottom panel shows human iTregs that were cotransfected with WT or mutant luciferase reporters with mimic miR-138 into HEK-293T cells, followed by assessment of relative luciferase activity. (**F**) Cotransfection of miR-138 and BCYRN1 in Tregs followed by assessments of ATG7 by WB. One-way ANOVA followed by Bonferroni’s post hoc test was used to determine statistical significance. All data are presented as mean ± SEM of 3 or 4 individual experiments (biological replicates). *P < 0.05, **P < 0.01, ***P < 0.001 versus control group.

ATG7-dependent autophagy is essential for the survival and proliferation of Tregs (25). To determine if CDC-EVs promote autophagy, we measured the protein levels of classic autophagic markers in human iTregs. As shown in Figure 4A, CDC-EVs increased ATG7 and MAP1LC3B, whereas SQSTM1, a marker of autophagic flux, was decreased, consistent with the notion that CDC-EVs induced autophagy. When applying EVs in which BCYRN1 had been suppressed by siRNA, autophagy induction was blunted (Figure 4B). Conversely, BCYRN1 overexpression increased autophagy markers in iTregs (Figure 4C). One way that lncRNAs, including BCYRN1, alter gene expression is by acting as “sponges” for specific miRNAs (26, 27). Because BCYRN1 is predicted to bind miR-138 (28), we used RNA pull down to test this prediction. After incubating biotin-labeled BCYRN1 probe with lysates of iTregs overexpressing BCYRN1, the precipitates were enriched in miRNA-138 (miR-138) (and BCYRN1, as a positive control), while negative controls *GAPDH* and *U6* were undetectable (Ct values >40; Figure 4D).

We further assessed the interactions between BCYRN1 and miR-138 by mutating the potential miR-138 binding site within BCYRN1 and gauged reporter gene expression. Cotransfection of the WT or mutant luciferase reporters with miR-138 into HEK-293 cells revealed that miR-138 reduced the activity of the WT reporter by >50% but had no effect on the mutant reporter (Figure 4E). ATG7 is a known target of miR‑138, as verified by luciferase assays (Supplemental Figure 1A; supplemental material available online with this article; https://doi.org/10.1172/JCI179262DS1). Indeed, ATG7 was suppressed by miR-138, an effect reversed by overexpressing BCYRN1 (Figure 4F). Thus, the data support the concept that BCYRN1 increases human iTreg proliferation by sponging miR-138 to induce ATG7-dependent autophagy.

### CDC-EV BCYRN1 induces CCR6-dependent migration by sponging miR-150.

**Figure 5:**
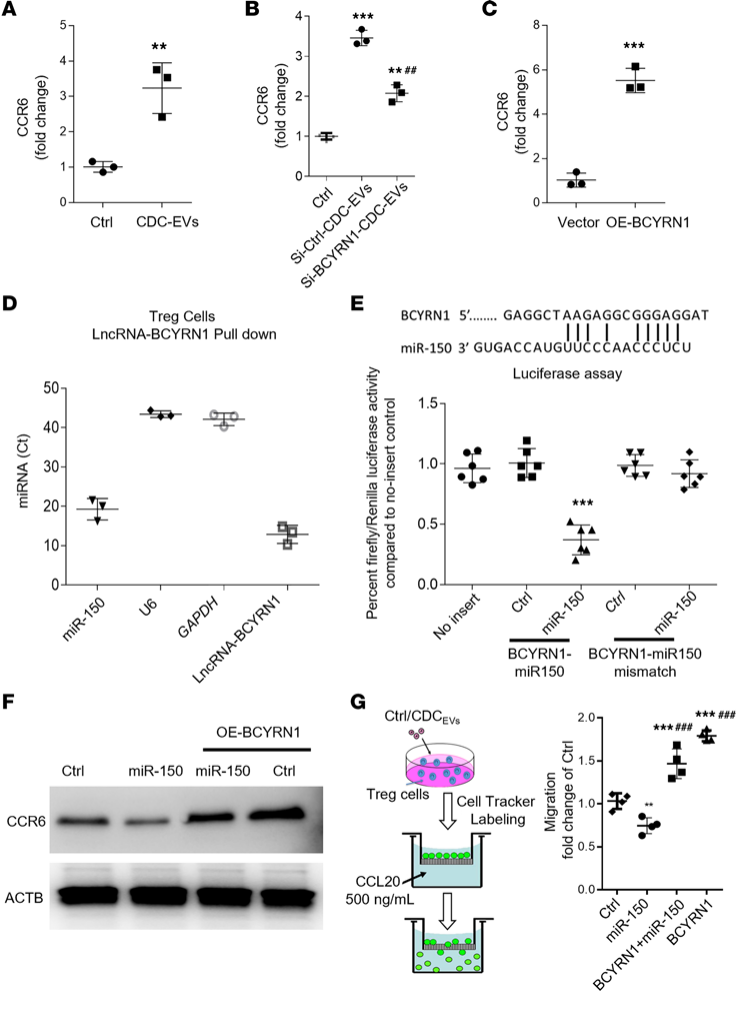
CDC-EV BCYRN1 induces Treg migration by competitively binding miR-150 to regulate CCR6 expression. (**A**) Human iTregs were exposed to CDC-EVs or were not treated (ctrl). The expression of CCR6 was assessed by qPCR. (**B**) Human Tregs were exposed to ctrl-CDC-EVs or si-BCYRN1 CDC-EVs (EV with BCYRN1 knockdown) or were not treated (ctrl). The expression of CCR6 was assessed by qPCR. (**C**) Human Tregs were transfected with vector or OE-BCYRN1 lenti-vector, followed by assessment of CCR6 by qPCR. (**D**) Biotin-labeled BCYRN1 probe was used to pull down BCYRN1-associated RNAs, followed by assessment of miR-150, negative control (U6 and GAPDH), and positive control (BCYRN1) expression by qPCR. (**E**) The top panels putative lncRNA BCYRN1 binding sites in miR-150. The bottom panel shows human iTregs that were cotransfected with WT or mutant luciferase reporters and mimic miR-150 into HEK-293T cells, followed by the assessment of relative luciferase activity. (**F** and **G**). Cotransfection of miR-150 and BCYRN1 in Tregs followed by assessments of CCR6 by WB (**F**) and cellular migration by trans-well migration assay (**G**). One-way ANOVA followed by Bonferroni’s post hoc test was used to determine statistical significance. All data are presented as mean ± SEM of 3 or 4individual experiments (biological replicates). **P < 0.01, ***P < 0.001 versus control group. ^###^*P* < 0.001 versus miR-150 group.

CCR6 recruits Tregs into inflammatory tissue (29, 30), and *CCR6* transcript levels were elevated in human iTregs exposed to CDC-EVs (Figure 5A). To probe the role of BCYRN1, we studied human iTregs exposed to either si-ctrl-CDC-EVs or si-BCYRN1-CDC-EVs (BCYRN1 knockdown in CDC-EVs). As shown in Figure 5B, si-ctrl-CDC-EVs (i.e., “normal” EVs) upregulated *CCR6*, but this effect was much reduced after suppression of BCYRN1 in human iTregs exposed to si-BCYRN1-CDC-EVs. Direct transfection with a BCYRN1-expressing lentiviral vector likewise increased *CCR6* transcript levels, but empty vector did not (Figure 5C). Results of analysis of lncRNA-miR interactions led us to predict that miR-150 is a sponge target of BCYRN1 (28); in turn, *CCR6* is a potential target of miR-150. RNA pull down with a biotin-labeled BCYRN1 probe showed miR-150 and BCYRN1 (positive control) were highly enriched in the precipitates, while negative controls *GAPDH* and *U6* were not detectable (Figure 5D). To further probe the interaction between BCYRN1 and miR-150, we mutated the binding site within BCYRN1 for miR-150 (31) and measured luciferase expression. While miR-150 reduced the activity of the WT reporter, the mutant reporter was not affected (Figure 5E). Consistent with an earlier report (32), luciferase assays validate that miR-150 indeed targets *CCR6* (Supplemental Figure 1B). Furthermore, overexpression of BCYRN1 attenuated miR-150’s ability to reduce CCR6 (Figure 5F), while boosting migration of human iTregs (Figure 5G). Taken together, the data demonstrate that BCYRN1 can sponge miR-150 to induce CCR6-dependent migration of human iTregs.

### CDC-EV BCYRN1 induces expression of IL-10 by sponging miR-98.

**Figure 6:**
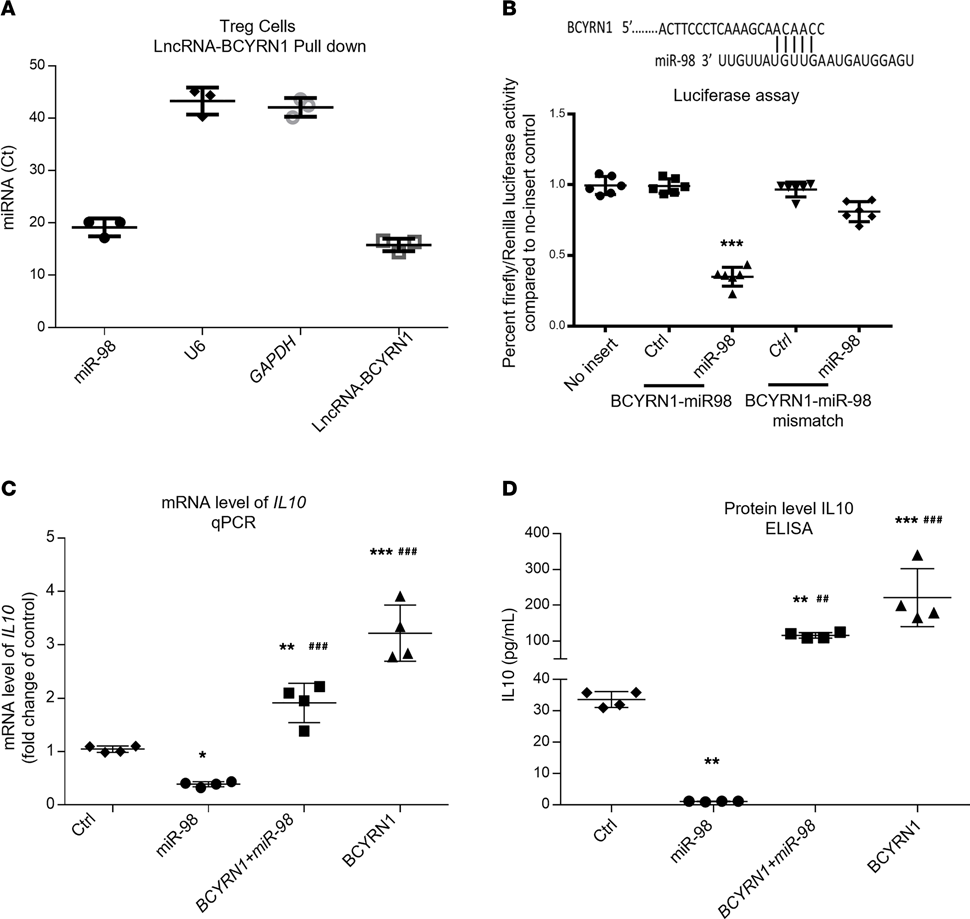
BCYRN1 mediates induction of IL-10 in Tregs by competitively binding miR-98 to regulate IL-10 expression. (**A**) Biotin-labeled BCYRN1 probe was used to pull down BCYRN1-associated RNAs, followed by assessment of miR-98, negative control (U6 and *GAPDH*), and positive control (BCYRN1) expression by qPCR. (**B**) Putative lncRNA BCYRN1 binding sites in miR-98 and luciferase assay results. (**C** and **D**) Cotransfection of miR-98 and BCYRN1 in Tregs, followed by assessments of IL-10 by qPCR (**C**) and ELISA (**D**). One-way ANOVA followed by Bonferroni’s post hoc test was used to determine statistical significance. All data are presented as mean ± SEM of 3 or 4 individual experiments (biological replicates). **P < 0.01, ***P < 0.001 versus control group. ^##^*P* < 0.01, ^###^*P* < 0.001 versus miR-150 group.

After analysis of lncRNA-miR interactions, we predicted that BCYRN1 could sponge miR-98 (28), a miR that, in turn, targets *IL-10* (33). RNA pull-down precipitates were enriched in miR-98 (and BCYRN1, as a positive control), while negative controls *GAPDH* and *U6* were undetectable (Ct values >40; Figure 6A). Additionally, in a luciferase reporter system, miR-98 reduced the activity of the WT reporter but not a mutant reporter in which the miR-98 binding site within BCYRN1 had been disrupted (Figure 6B). Furthermore, IL-10 was suppressed by miR-98, an effect reversed by overexpressing BCYRN1 (Figure 6, C and D). To demonstrate binding between miR-98 and its target (*IL10*), we conducted luciferase assays (Supplemental Figure 1C). Next, we asked whether the BCYRN1 construct with the disrupted miR-98 binding site (mut-BCYRN1) still suppressed IL-10 levels. As shown in Supplemental Figure 2, A and B, WT BCYRN1 enhances IL-10 expression at both the mRNA (by qPCR) and protein levels (by ELISA) in Tregs. In contrast, mut-BCYRN1, which contains a disrupted binding site for miR-98, does not augment IL-10, indicating that an intact miR-98 binding site in BCYRN1 is crucial for its ability to modulate IL-10. Further validating this mechanism, Supplemental Figure 2, C and D, reveals that miR-98 suppressed IL-10 expression, but this suppression could be reversed by overexpressing WT BCYRN1 (but not mut-BCYRN1). Thus, BCYRN1 increases expression of IL-10 in human iTreg by sponging miR-98.

### Therapeutic efficacy of CDC-EVs and CDC-EV-BCYRN1 in MI.

**Figure 7:**
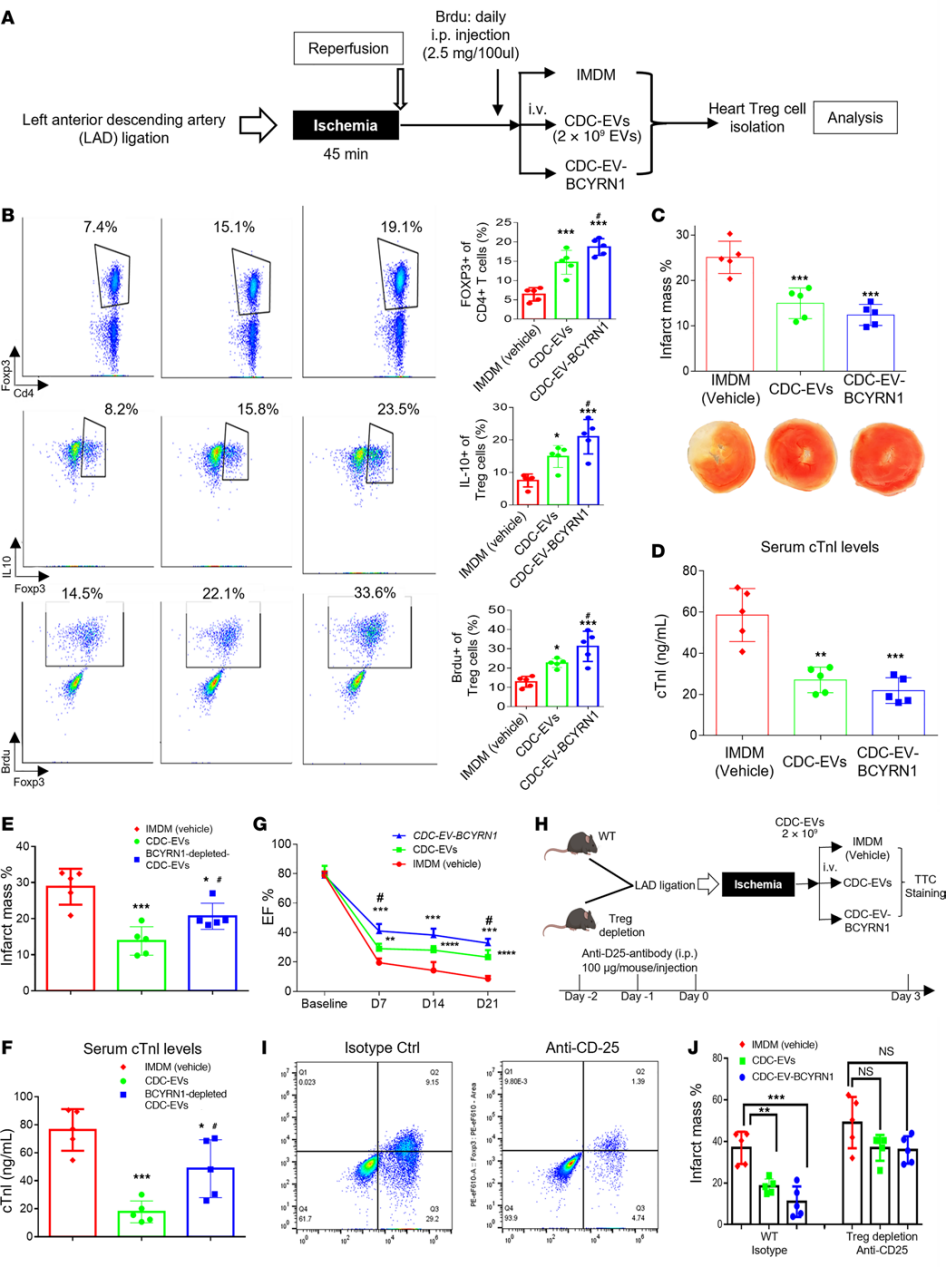
Therapeutic efficacy of CDC-EVs and CDC-EV-BCYRN1 in a mouse myocardial infarction model — the role of Tregs. (**A**) Schematic representation of in vivo myocardial infarction (MI) protocol. (**B**) Representative flow cytometry plots and pooled data of the CD4^+^Foxp3^+^, CD4^+^Foxp3^+^IL-10^+^, and CD4^+^Foxp3^+^Brdu^+^ populations in hearts from animals infused with CDC-EVs, CDC-EVs overexpressing BCYRN1 (CDC-EV-BCYRN1), and IMDM (vehicle) (*n* = 5 mice per group). (**C**) Pooled data for percentage of infarct mass (*n* = 5 mice per group) and representative images of TTC-stained hearts from CDC-EV–, CDC-EV-BCYRN1–, and vehicle-injected animals 72 hours after MI. (**D**) Plasma cTnI values from CDC-EVs, CDC-EV-BCYRN1– and vehicle-injected animals (*n* = 5/group) 24 hours after MI. (**E**) Pooled data for percentage of infarct mass (*n* = 5/group) of TTC-stained hearts from CDC-EV–, BCYRN1-depleted CDC-EV–, and vehicle-injected animals 72 hours after MI. (**F**) Plasma cTnI values from CDC-EV–, BCYRN1-depleted CDC-EV–, and vehicle-injected animals (*n* = 5/group) 24 hours after MI. (**G**) Echocardiographic analysis of left ventricular ejection fraction (EF) on days 0 (baseline before MI), 7, 14, and 21 after MI with indicated treatment (*n* = 5/group). (**H**) Schematic of Treg depletion and in vivo ischemia/reperfusion (I/R) protocol. Mice were injected twice with anti-CD25 antibody (100 μg/mouse/injection i.p.), or isotype control daily for 2 days before I/R. On day 3, TTC assay was used to assess infarct size (*n* = 5/group). (**I**) Representative plots showing the percentage of CD25^+^FoxP3^+^ in CD4^+^ T cells (Q2 quadrant). (**J**) Quantitative measurements of the percentage of infarct mass (*n* = 5/group) from CDC-EV– or CDC-EV-BCYRN1–injected animals (WT/Treg depletion) at 72 hours after I/R injury. One-way ANOVA followed by Bonferroni’s post hoc test was used to determine the statistical significance among multiple groups. *P < 0.05, **P < 0.01, ***P < 0.001, ****P < 0.0001 versus control group. ^#^*P* < 0.05 versus CDC-EVs group.

CDC-EVs reduce infarct size in rats after MI (20, 34). Here, we have demonstrated BCYRN1 is plentiful in CDC-EVs, and, on its own, BCYRN1 can upregulate human iTregs. Thus, we tested the hypothesis that an increase in Tregs underlies the cardioprotective effects of CDC-EVs in MI, due, at least in part, to BCYRN1 in the CDC-EV cargo. Fifteen minutes after MI, mice received i.v. CDC-EVs, CDC-EVs overexpressing BCYRN1 (CDC-EV-BCYRN1), or vehicle (IMDM; Figure 7A). Infarct mass, circulating TnI levels, and infiltrating Tregs were assessed 72 hours later. In mice given CDC-EVs, the number of Cd4^+^Foxp3^+^IL-10^+^ cells was increased and was even higher in mice that received CDC-EV-BCYRN1, relative to vehicle controls (Figure 7B). Thus, CDC-EVs and CDC-EV-BCYRN1 promoted Treg infiltration into the heart and augment IL-10–producing Tregs. Additionally, CDC-EVs, and CDC-EV-BCYRN1, increased the number of Cd4^+^Foxp3^+^Brdu^+^ cells (i.e., proliferating Tregs) in the heart. Either CDC-EVs or CDC-EV-BCYRN1 significantly decreased infarct mass and serum TnI levels compared with vehicle control (Figure 7, C and D). The extent of scar reduction was so large (≥50% relative reduction) that it is perhaps not surprising that the cardioprotective effects of either intervention (viz., CDC-EVs with or without enhanced BCYRN1 expression) were comparable, as there was little room for further improvement.

To further probe the role of BCYRN1 in CDC-EVs in vivo, we knocked down BCYRN1 in CDC-EVs to verify loss of cardioprotective function. Figure 3, D and E, shows that transfection of CDCs with siRNA-BCYRN1 decreased the expression of BCYRN1 in both CDCs and CDC-EVs. Thus, we transfected CDCs with either si-ctrl or si-BCYRN1 for 48 hours, followed by EV isolation, and studied the effects of the EVs in vivo. Fifteen minutes after MI, mice received i.v. si-ctrl-CDC-EVs (EVs isolated from CDCs exposed to siRNA control), BCYRN1-depleted CDC-EVs, or vehicle. Pooled data showed that CDC-EVs decreased infarct mass (Figure 7E) and serum levels of cardiac troponin I (cTnI) (Figure 7F), confirming earlier findings (cf., Figure 7C), but these cardioprotective effects were abrogated when BCYRN1 was depleted in CDC-EVs (Figure 7, E and F). The reductions of infarct size by CDC-EVs and CDC-EV-BCYRN1 translated into significant functional improvements in vivo: both treatments enhanced ejection fraction (EF) relative to that in the control group (Figure 7G). Notably, while CDC-EVs led to a moderate improvement in EF, CDC-EV-BCYRN1 treatment resulted in even more substantial increases in EF. Thus, enhanced levels of BCYRN1 confer greater functional recovery, underscoring the added therapeutic benefit of supplementing BCYRN1 in CDC-EVs. Thus, BCYRN1 plays a crucial role in CDC-EVs’ disease-modifying bioactivity.

In a complementary approach, we depleted Tregs by injection of anti-CD25 antibody to achieve approximately 80% Treg depletion (Figure 7I). WT (i.e., nondepleted) mice received isotype control (rat IgG1). Afterward, CDC-EVs or CDC-EV-BCYRN1 were administered i.v. 15 minutes after reperfusion (as in Figure 7H). Hearts were excised 72 hours after MI followed by 2,3,5-Triphenyl-2H-tetrazolium chloride (TTC) staining to quantify infarct mass histologically. Pooled data (Figure 7J) show that both CDC-EVs and CDC-EV-BCYRN1 decreased infarct mass, and this protective effect was abrogated when Tregs were depleted. Thus, Tregs are required for CDC-EVs’ and CDC-EV-BCYRN1’s disease-modifying bioactivity. The small (not statistically significant) benefits of CDC-EVs and CDC-EV-BCYRN1 after Treg depletion, if real, would be consistent with the idea that cells other than Tregs (e.g., macrophages) might be minor effectors of CDC-EV– and CDC-EV-BCYRN1–mediated cardioprotection.

## Discussion

MI is a leading cause of death in the United States (35). Tregs can potentially promote tissue repair and functional recovery after MI (36), but it is difficult, costly, and time-consuming to isolate and expand Tregs ex vivo for clinical use (7). A therapy that can selectively and quickly expand and activate Tregs in vivo is highly desirable. CDC-EVs are known to increase the proliferation of Tregs and augment their production of IL-10 (12), but, until now, the mechanism underlying these salutary effects was unclear. Here, we have pinpointed BCYRN1, a lncRNA plentiful in CDC-EVs, as responsible for expanding and activating Tregs. Acting as a miR sponge, BCYRN1 suppresses miR-138 (targeting ATG7-dependent autophagy), miR-150 (targeting CCR6-dependent migration), and miR-98 (targeting IL-10) to enhance Treg proliferation, migration, and IL-10 production in vitro and in vivo. These salutary effects, in turn, lead to cardioprotection against MI.

Genetic therapy, especially with RNA-based drugs, is becoming a promising strategy for the treatment of human diseases that are refractory to conventional approaches (37). Most RNA drugs are targeted therapies — such as siRNA, antisense RNA, and aptamers (38) — but these are only the tip of the iceberg for RNA-based approaches. Here, we take a complementary approach: mining EVs from therapeutically active cells to identify ncRNA lead compounds. This line of investigation began with CDCs, which are now in advanced clinical testing for Duchenne muscular dystrophy (10). When our mechanistic work revealed the beneficial effects of CDCs to be indirect (39, 40), the focus logically shifted to understanding and optimizing the mediators. EVs, including exosomes, microvesicles, and apoptotic bodies, vary widely in size. Exosomes, which are of particular interest, typically range from 30 to 150 nm in diameter. Using a 100 kDa filter to concentrate vesicles within this size range helps to ensure that the isolated vesicles are predominantly exosomes or small microvesicles (41). Filtration using a 100 kDa filter is a relatively simple, fast, and efficient method of EV isolation compared with other techniques, such as ultracentrifugation or size-exclusion chromatography. Using this protocol, we consistently isolated CDC-EVs with a modal size of approximately 131 nm, and such EVs richly expressed exosome markers (Supplemental Figure 3, A and B). CDC-EVs (19) contain many molecularly distinct ncRNA entities with known or plausible bioactivity. CDC-EV cargo contains a diversity of ncRNAs, including miRs, Y RNAs, tRNAs, and the lncRNA BCYRN1, some of which have previously unrecognized bioactivity (42). Here, we have implicated BCYRN1 as a determinant of Treg proliferation and activation. The presence of multiple types of ncRNA within the same EV preparations, with complementary mechanisms of action, sets up these molecules to act synergistically or additively to enhance cardiac protection. However, this heterogeneity also means that the individual contributions of different ncRNAs may vary depending on their relative abundance and the specific context of their interactions within the heart after MI.

Recently, lncRNAs have emerged as potential modulators of gene expression in diverse cell lineages, with broad-ranging bioactivity (43). Various lncRNA isoforms act by binding and sequestering selected miRNAs (“miR sponge” mechanism), thereby releasing functional targets from miR translational suppression. These features of lncRNA play a crucial role in immune cells, which demonstrate dynamic functional plasticity in response to their local microenvironments (44). Here, we found one particular species — BCYRN1 — is highly enriched, being the most plentiful lncRNA in CDC-EVs. BCYRN1 was first described in the developing and adult nervous system, where it is actively trafficked to dendrites to regulate synaptogenesis (45–48). Emerging evidence further implicates BCYRN1 in promoting cell proliferation and survival (49). However, there are no prior studies in heart cells or their EVs, and no one has previously reported effects of BCYRN1 on Tregs. The rationale for selecting miR-138, miR-150, and miR-98 to investigate the effect of BCYRN1 is based on comprehensive preliminary studies and bioinformatics analyses that highlighted their potential roles in Treg function. Our earlier research established that CDC-EVs enhance Treg proliferation and induce IL-10 production (12). Functional heatmap analyses (Figure 1, B and C) based on IPA revealed an enrichment of cell survival and cellular movement signaling in the transcripts upregulated by CDC-EVs compared with vehicle, indicating that CDC-EVs promote Treg migration as well. Further investigation showed that BCYRN1 is highly enriched in CDC-EVs, leading us to probe its potential role in inducing or influencing Treg proliferation, migration, and IL-10 production. Given the well-documented role of lncRNAs as miRNA sponges, we conducted a targeted bioinformatics analysis focusing on how BCYRN1 might sponge miRNAs that regulate critical Treg functions. Our lncRNA-miRNA association analysis identified 3 miRNAs — miR-138, miR-150, and miR-98 — as being potentially bound by BCYRN1 (Figure 4E, Figure 5E, and Figure 6B). These miRNAs have been linked to relevant Treg biology: (a) miR-138 targets and suppresses ATG7, which is vital for Treg survival and proliferation (50); (b) miR-150 targets CCR6, which facilitates the migration of Tregs into inflammatory tissues (29, 32); and (c) miR-98 regulates IL-10, an antiinflammatory cytokine produced by Tregs (33). Thus, the selection of these specific miRNAs was driven by their established roles in pathways critical to Treg function and survival.

We found that CDC-EV BCYRN1 could induce proliferation of human iTregs via sponging miR-138, enhancing ATG-7–dependent autophagy. A cellular process of self-degradation, autophagy regulates various components of the immune system, including natural killer cells, macrophages, dendritic cells, and T and B lymphocytes (51). ATG7 helps form autophagosomes; interestingly, Tregs lacking ATG7 exhibit increased apoptosis and rapidly lose expression of Foxp3, particularly following activation (50). We found that miR-138 targets ATG7, inhibiting autophagy and thereby reducing iTreg proliferation. Overexpression of BCYRN1 attenuated miR-138–mediated downregulation of ATG7, promoting proliferation. The IL-33/ST2 axis, which modulates the accumulation and expansion of heart Tregs (52), intersects with ATG7-dependent autophagy to enhance degradation of Beclin1-associated factors, favoring tissue healing (53). Additionally, IL-33 elevates the expression of ATG5 and Beclin1 while inhibiting SQSTM1 during Treg expansion, consistent with the notion that expansion of Tregs by IL-33 is associated with its regulation of autophagy (54). Thus, BCYRN1-induced ATG7-dependent autophagy and the IL-33/ST2 axis may synergistically augment Treg expansion and function in the heart.

Tregs, like all other lymphocytes, migrate to sites of inflammation to carry out their regulatory functions. The expression of specific chemokine receptors on the surface of Tregs facilitates their homing. Chemokine CCL20 has been linked to ischemic heart disease, specifically acute MI (55), insofar as levels of CCL20 in the blood of patients with MI are higher than those in healthy individuals (56). The receptor for CCL20, CCR6, plays a crucial role in the recruitment of Tregs to the ischemic heart (29). We found that CDC-EVs induced upregulation of CCR6 and Treg migration, effects that were enhanced by overexpression of BCYRN1. RNA pull-down and luciferase assays revealed that BCYRN1 acts as a sponge for miR-150. While miR-150 suppressed CCR6 protein and migration of human iTregs, overexpression of BCYRN1 reversed this suppression. In short, we have demonstrated that CDC-EVs induce CCR6-dependent migration of Tregs via BCYRN1, which acts as a sponge for miR-150. The increase in cell migration may facilitate enhanced infiltration of injured cardiac tissue by Tregs.

IL-10, a pleiotropic cytokine that plays a crucial role in the regulation of immune responses, is produced by Tregs, monocytes, Th2 cells, subsets of activated T cells, and B cells (57). Treg-derived IL-10 plays a vital role in maintaining immunotolerance by preserving FOXP3 expression, stability, and associated regulatory mediators (58). CDC-EVs increase IL-10 production by Tregs to dampen systemic and local inflammation in myocarditis models. Although Tregs suppress inflammation through multiple factors, such as TGFB, IL35, CTLA4, and CD25, our previous study demonstrated that only the expression of IL-10 increased after CDC-EV treatment. This suggests that the beneficial effects we found in vitro result from elevated levels of IL-10 (12). Using gene knockdown and overexpression approaches, we implicated BCYRN1 as the critical player in CDC-EV–mediated induction of IL-10 in human iTregs. IL-10 upregulation can be explained by BCYRN1’s ability to act as a sponge for miR-98, rationalizing how CDC-EVs induce IL-10 in human iTregs.

Indeed, miRs regulate a wide array of target proteins. We specifically focused on miR-138, -150, and -98 due to their roles in regulating key proteins like ATG7, CCR6, and IL-10. These targets are crucial for Treg functions, such as survival, migration, and antiinflammatory responses, directly aligning with the objectives of our research on BCYRN1 in cardiac therapy. These miRNAs were selected based on strong evidence from preliminary studies and detailed bioinformatics analyses. This targeted approach helps us understand and enhance BCYRN1’s potential in treating heart conditions. Regarding the evaluation of potential off-target effects, we acknowledge that this merits further exploration, but such studies are beyond the scope of the present work.

Modulating Tregs is a promising therapy for inflammatory diseases, utilizing the body’s natural immune suppression mechanisms. More than 50 clinical trials (59) are currently exploring Treg autotransfusion for conditions, including solid organ transplantation (to increase graft survival), graft-versus-host disease, and autoimmune disorders. Most of these trials use a lengthy, complex protocol that involves isolating and expanding Tregs ex vivo before reinfusing them back into the patient. Not only does this introduce uncertainty regarding Tregs’ suppressive and growth capabilities after expansion, but this also involves long delays that obviate applicability to acute disease, such as MI or stroke. Our in vivo data demonstrate that CDC-EVs and CDC-EV-BCYRN1 could induce IL-10^+^ Treg infiltration into the heart, which, in turn, reduced infarct size and decreased concentrations of the ischemic biomarker cTnI. Prior experimental assessments (12) also suggested specificity for Tregs (i.e., no change in Th1/Th2 but a modest inhibition of Th17 cells). In further investigating effects on T cell subsets, we found that treatment with CDC-EVs and CDC-EV-BCYRN1 resulted in a decrease in the proliferation of Th1 and Th17 cells, while Th2 cell proliferation remained unaffected (Supplemental Figure 4). This observation aligned with our in vitro data (Supplemental Figure 5), where overexpression of BCYRN1 in mouse Th1, Th2, Th17, and Tregs led to increased proliferation across these types, albeit differentially. Notably, the fold change in Treg proliferation was significantly higher than in other T cell subsets, suggesting a more pronounced role of BCYRN1 in promoting Treg proliferation. Thus, while BCYRN1 overexpression generally supports T cell proliferation, its most substantial impact appears to be on Tregs. The enhanced expansion of Tregs could potentially contribute to the suppression of Th1 and Th17 proliferation in vivo, a mechanism that might explain the differential proliferation rates observed after treatment in our MI model. This differential response underscores the complexity of immune modulation by BCYRN1 and supports the specificity of Treg targeting as a promising therapeutic strategy in MI. The success of this approach is particularly notable given that the EVs were administered 15 minutes after reperfusion. Such timing is compatible with standard clinical practice: given the paramount importance of recanalization of the culprit vessel, adjunctive therapy cannot realistically be applied until after reflow has been established (60).

While the potential utility of Tregs in mitigating cardiac injury has long been recognized (52, 61–63), clinical translatability has been undermined given the lack of a viable approach to upregulate Tregs in the acute setting. The approach of using CDC-EVs, or CDC-EV-BCYRN1, to harness Tregs in vivo bypasses the limitations of ex vivo expansion. This discovery may lead to the development of an RNA-based approach targeting Treg function as an immunomodulatory therapy suitable for acute illness. While here we have shown disease-modifying bioactivity in MI, the potential utility of BCYRN1 to boost Tregs is in no way limited to the heart; identical or analogous approaches merit testing in a broad variety of inflammatory diseases where increased Treg activity is a logical therapeutic goal.

## Methods

### Sex as a biological variable.

Male mice were chosen because they are known to exhibit more severe manifestations of MI than female mice, including worse survival, functional recovery, and remodeling (64).

### Animals.

Male 10- to 12-week-old Foxp3-IRES-mRFP mice, purchased from The Jackson Laboratory, were used for in vivo experiments.

### MI mouse model.

MI was induced (20) via a left parasternal thoracotomy performed under anesthesia. A ligature was tightened around the left anterior descending coronary artery for 45 minutes of ischemia and then released to allow reperfusion. Fifteen minutes later, we administered an i.p. injection of BrdU (2.5 mg/100 μL) 1 hour prior to the i.v. injection of 100 μL of each test item (EV-BCYRN1 or CDC-EVs, 2 × 10^9^ EVs/ mouse) or vehicle (IMDM).

### CD25 antibody–mediated Treg depletion.

When so indicated, 2 days prior to MI, Tregs were depleted by daily i.p. injection of anti-CD25 antibody (100 μg/mouse) (65). As a control, nondepleted mice were given isotype control (rat IgG1) by the same method and timing.

### Cell culture.

CDCs were isolated as described previously (66). Briefly, human heart tissue was chopped into fragments and enzymatically digested with collagenase. Tissue was then cultured as explants on fibronectin-coated flasks (Corning, 356009) at 37°C, in 5% CO_2_, 5% O_2_-equilibrated IMDM with 20% FBS and penicillin (100 U/mL). After 2–3 weeks, an outgrowth of stromal-like cells and phase-bright round cells from the tissue fragments reached 80% confluence. These cells were then harvested and seeded onto Ultra-Low Attachment flasks (Corning, CLS3814) to support cardiosphere formation. Two days later, CDCs were formed by seeding cardiospheres on fibronectin-coated flasks and cultured in IMDM with 20% FBS and expanded to passage 4–6 for EV isolation.

Human iTregs were purchased from IQ Biosciences (IQB-Hu1-iTr-1) and expanded (Treg Expansion Kit, Miltenyi Biotec, 130-095-353) according to the manufacturer’s protocol. Human Tregs were used within 5 passages.

### EV isolation.

As described, EVs were prepared from FBS-depleted media conditioned by CDCs using ultrafiltration by centrifugation (12). Briefly, 15 day–conditioned media were harvested and centrifuged at 1,000*g* for 10 minutes to eliminate cells, followed by filtration through a 0.22 μm filter to remove cell debris. Then, the CDC-EVs were concentrated using 100 kDa filters (Millipore) at 3,000*g* for 30 minutes. EV size and number were assessed using a nanoparticle tracking system.

### Overexpression of BCYRN1 in human iTregs.

For lentiviral vector overexpressing BCYRN1 (Applied Biological Materials Inc.), the full-length BCYRN1 cDNA was inserted into the pLenti-CMV-GFP-2A-Puro vector (Applied Biological Materials Inc.) at EcoRV sites, and BCYRN1 expression was driven by a CMV promoter. In our study, human iTregs (4 × 10^5^ cells/well) were transfected with 1 μg lentiviral BCYRN1 vector in a 24-well format using DharmaFECT Transfection Reagent (T-2006-01) at 3 μL per well. An empty vector (pLenti-CMV-GFP-2A-Puro) served as a negative control.

### Cell proliferation assay.

Cell proliferation was assessed using Cell Counting Kit-8 (Sigma-Aldrich) according to the manufacturer’s instructions (67). Briefly, human iTregs were seeded in 96-well plates at a density of 5,000 cells/well, followed by addition of CDC-EVs (1,000 particles/cell), transfecting lentiviral BCYRN1 vector or empty lentiviral vector for 72 hours. Then, 10 μL CCK-8 solution (water-soluble tetrazolium salt [WST-8]) was added into each well for 4-hour incubation. WST-8 was reduced by dehydrogenase activities in living cells to give a yellow color formazan dye. Afterward, spectrophotometric absorbance was measured at 450 nm for each well. Experiments were repeated 3 times, with each sample in triplicate. Pooled data are presented as mean ± SEM.

### Transwell migration.

Boyden chambers (Corning Costar) were used to assess the transmigration of Tregs (68). Briefly, human iTregs were seeded (5 × 10^5^ cells/well) onto 6.5 mm Transwell inserts with PET membrane (Costar, 3464). Culture medium (600 μL) with 500 ng/mL recombinant mouse CCL20 was placed in the lower chamber. The Transwell plates were incubated for 5 hours at 37°C, followed by quantification of migrated Tregs in the lower chamber. Data represent results from 3 biological replicates, and each biological replicate comprised 2–3 technical replicates.

### Luciferase activity assays.

A 40 bp/44 bp lncRNA BCYRN1 3′UTR segment containing the putative miR-150 target site (sense 5′-AAACTAGCGGCCGCTAGTGAGGCTAAGAGGCGGGAGGATT-3′ and antisense 5′-CTAGAATCCTCCCGCCTCTTAGCCTCACTAGCGGCCGCTAGTTT-3′), miR-138 target site (5′-AAACTAGCGGCCGCTAGTTCCCTCAAAGCAACAACCCCCT-3′ and antisense 5′- CTAGAGGGGGTTGTTGCTTTGAGGGAACTAGCGGCCGCTAGTTT-3′), and miR-98 target site (sense 5′-AAACTAGCGGCCGCTAGTACTTCCCTCAAAGCAACAACCT-3′ and antisense 5′-CTAGAGGTTGTTGCTTTGAGGGAAGTACTAGCGGCCGCTAGTTT) was cloned into the PmeI and XbaI sites of the pmirGLO vector (Promega, E1330). For pmirGLO-BCYRN1 3′UTR-miR-138–target-mutant segment (sense 5′-AAACTAGCGGCCGCTAGTTCCCTCAAAGCATGTTGCCCCT-3′ and antisense 5′-CTAGAGGGGCAACATGCTTTGAGGGAACTAGCGGCCGCTAGTTT-3′), the miR-138 target site (ACAAC) within the BCYRN1 3′UTR was changed to (TGTTG), for pmirGLO-BCYRN1 3′UTR-miR-150-target-mutant segment (sense 5′-AAACTAGCGGCCGCTAGTGAGGCTTTCACGCCCCTCGATT-3′ and antisense 5′-CTAGAATCGAGGGGCGTGAAAGCCTCACTAGCGGCCGCTAGTTT-3′), the miR-150 target site (GGGAG) within the BCYRN1 3′UTR was changed to (CCCTC), and for pmirGLO-BCYRN1 3′UTR-miR-98-target-mutant segment (sense 5′-AAACTAGCGGCCGCTAGTACTTCCCTCAAAGCATGTTGCT-3′ and antisense 5′-CTAGAGCAACATGCTTTGAGGGAAGTACTAGCGGCCGCTAGTTT-3′), the miR-98 target site (ACAAC) within the BCYRN1 3′UTR was changed to (TGTTG). Following the manufacturer’s protocol (Promega), Tregs were seeded into 24-well plates. Two days after cotransfecting Tregs with miR-138, miR-98, and miR-150 mimic or a mimic RNA negative control, along with either a pmirGLO-BCYRN1-3′UTR-miRNA-target or pmirGLO-PTEN-3′UTR-BCYRN1-miRNA target-mutant luciferase reporter vector using DharmaFECT 1 Transfection Reagent (PerkinElmer), luciferase activity was measured using the Dual-Luciferase Reporter Assay (Promega, E2940). Firefly luciferase activity was normalized by renilla luciferase activity and expressed as a percentage of control. Experiments were performed in triplicate, with 3 wells each time.

### Flow cytometry.

The following antibodies were used for staining: Brilliant Violet 421 anti-mouse Cd4 Antibody (clone GK1.5; BioLegend), PE anti-mouse Cd25 Antibody (PC61; BioLegend), Alexa Fluor 594–conjugated anti-Mouse Foxp3 Antibody (clone 1054C; R&D Systems), APC/Cyanine7 anti-mouse Cd45 Antibody (clone 30-F11; BioLegend), and Alexa Fluor 700–conjugated anti-IL-10 (clone JES5-16E3; eBioscience). Viability staining was performed with Zombie Aqua (BioLegend), and proliferation assays were performed (In Vivo EdU Flow Cytometry Kit 647, Sigma-Aldrich). According to the manufacturers’ protocols, the transcription factor staining buffer set (eBioscience) or intracellular fixation and permeabilization buffers (BioLegend) were used for intracellular staining of IL-10 and Foxp3. Samples were acquired using the Sony SA3800 spectral analyzer (Sony Biotechnology) and analyzed with FlowJo Software (BD Biosciences).

### TTC staining.

Three days after MI, hearts were arrested in diastole (10% KCl), excised, PBS washed, and cut into serial sections of ≈1 mm thickness. The sections were immersed in TTC (1% solution in PBS) for 15–20 minutes in the dark at 37°C. Surviving tissue turned deep red, while necrotic tissue remained pale. Once the color was established, the sections were fixed in 4% formalin for approximately 20 minutes followed by PBS wash. Then, the sections were imaged and weighed.

### ELISA assays.

Concentration of cTnI in serum was measured by a mouse-specific ELISA (Life Diagnostics, CTNI-1-HSP) according to the manufacturer’s instructions. Culture supernatants were collected from human Tregs exposed to various treatments, and IL-10 was quantified using the human IL-10 Elisa kit (Thermo Scientific, KHC0101C) according to the manufacturer’s instructions.

### Western blotting.

At the end of each protocol, human Tregs were lysed using RIPA buffer (Fisher Scientific, PI89900), as described previously (69), equal amounts of the proteins were electrophoresed in an SDS-polyacrylamide gel under reducing conditions followed by transfer to PVDF membranes. Blots were blocked with 3% BSA in TBS-Tween 20, and probed with antibodies specific for ATG7 (1:1,000; Novus Biologicals, MAB6608), MAP1LC3B (1:1,000; Novus Biologicals, NB100-2220), SQSTM1 (1:1,000; MBL International, PM045), and ACTB (1:5,000; Thermo Scientific, MA5-15739-HRP). Secondary antibodies were alkaline phosphatase conjugated to goat anti-mouse/rabbit IgG (1:10,000; Jackson ImmunoResearch Labs). Membranes were washed and incubated with an ECL solution and signals were detected using Supersignal West Dura Extended Duration or Pico PLUS Chemiluminescent Substrate (Thermo Fisher Scientific) and imaged on a Bio-Rad ChemiDoc imaging system. All experiments had at least 3 biological replicates; representative blots are shown in the figures.

### RNA preparation and next-generation sequencing and analysis.

EV RNA and Treg RNA were extracted in QIAzol (QIAGEN) and isolated using the RNeasy Mini Kit (QIAGEN) per the manufacturer’s instructions. Total RNA yields were quantitated by NanoDrop A260 for overall recoveries. Total RNA was assessed for quality, enriched, fragmented, ligated with adapters, and converted to cDNA. The cDNA was barcoded and amplified, and the RNA-Seq libraries were assessed. Raw sequencing data were multiplexed and processed into FASTQ format using bcl2fastq v2.20 (Illumina). Reads of EV RNA-Seq were then aligned to a comprehensive ncRNA database and annotated (20). Reads of Treg RNA-Seq were aligned to the mouse GRCm38 transcriptome using STAR/RSEM. A custom reference, including protein-coding and ncRNA genes, was downloaded from http://www.gencodegenes.org Gene expression counts were normalized using a modified trimmed mean of the M values normalization method. Wald’s test was used to assess the differential expressions between 2 sample groups by DESeq2 (12). Genes with an absolute log_2_ fold change (|log_2_ fold change|) higher than 2.5 and *P* values lower than 0.05 compared with the control samples were defined as differentially expressed and were analyzed by the IPA software (version 2021; Ingenuity Systems; QIAGEN). Differentially expressed genes from RNA-Seq outcomes were uploaded to IPA for disease functions analysis and heatmap generation.

### qPCR.

For quantitative analysis of mRNA and miRNA expression, comparative real-time PCR was performed with the use of Taqman Universal PCR Master Mix (Applied Biosystems). Specific primers and probes for *IL10*; *CCR5*; *CCR6*; *CCR7*; lncRNA BCYRN1; *GAPDH*; mature miR-138, -150, and -98; and small nuclear RNA RNU6B (*U6*) were obtained from Applied Biosystems. All reactions were run in triplicate. The relative expression levels of miRNA were calculated after normalization to small nuclear RNA RNU6B, with values expressed relative to the control group (70).

### Echocardiography.

To evaluate the effect of our treatment on cardiac functional recovery, echocardiography was conducted at days 0 (baseline before MI), 7, 14, and 21 after MI. Mice were anesthetized with isoflurane during examinations using a high-frequency ultrasound system (VisualSonics Vevo 2100). Images were analyzed using Vevo LAB software. Left ventricular EF was quantified to evaluate global heart function.

### Statistics.

Statistical analyses were performed using GraphPad Prism version 6 for Windows. A 2-tailed Student’s *t* test was employed for comparison between 2 groups. For multiple comparisons, 1-way ANOVA with a Bonferroni’s post hoc test was utilized. To determine the equality of variances between 2 groups, an F test was applied. For comparison among multiple groups, a Brown-Forsythe test was used to evaluate the equality of variances. Statistical significance was determined at *P* < 0.05. While data distribution was assumed to be normal, this assumption was not formally tested.

### Study approval.

All animal procedures were performed according to protocols approved by the IACUC at Cedars-Sinai Medical Center (IACUC 009183). Human heart tissue was obtained under an IRB-approved Pro00014713at Cedars-Sinai Medical Center. Written informed consent was obtained for the use of these human samples.

### Data availability.

The data underlying this article will be shared on reasonable request to the corresponding author. Values for all data points shown in graphs are reported in the Supporting data values file. The sequencing data have been deposited and can be accessed at https://github.com/Ke-Liao/BCYRN1-Treg-sequencing-Raw-data “(commit ID, 1797d70995db73f54ae50a50bb263490a69527ce)”.

## Author contributions

KL and EM established the hypotheses, designed the experiments, analyzed data, and wrote the manuscript. KL, JY, AA, ZM, LL, WL, and NA performed the experiments. AA and KL assisted with gene expression profile and performed bioinformatics analysis. AGEI obtained the RNAseq data on EVs and assisted in the analysis of these data.

## Supplementary Material

Supplemental data

Unedited blot and gel images

Supporting data values

## Version 1 03/25/2025

In-Press Preview

## Version 2 05/01/2025

Electronic publication
