# Supplementary material for: Long noncoding RNA BCYRN1 promotes cardioprotection by enhancing human and murine regulatory T cell dynamics
Source: J Clin Invest. 2025 Mar 25;135(9):e179262. doi: 10.1172/JCI179262 (PMC12043100; doi:10.1172/JCI179262)
Supplement: Supplemental data [file jci-135-179262-s008.pdf]

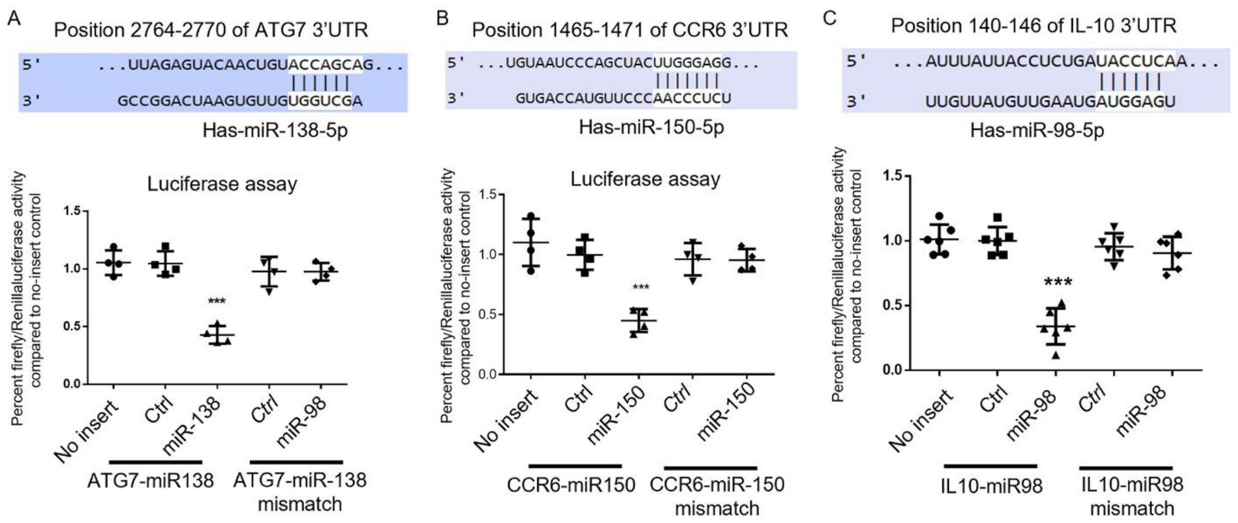

Figure S1 validation of miR-138, miR-150, and miR-98 target ATG7, CCR6, and IL10.

(A-C) Co-transfected wild type or mutant luciferase reporters with mimic miR-138 (A), miR-150 (B), and miR-98 (C) into HEK-293T cells, followed by the assessment of relative luciferase activity. One-way ANOVA followed by Bonferroni's post hoc test was used to determine the statistical significance among multiple groups. *, $P < 0.05$, **, $P < 0.01$, ***, $P < 0.001$ versus control group.

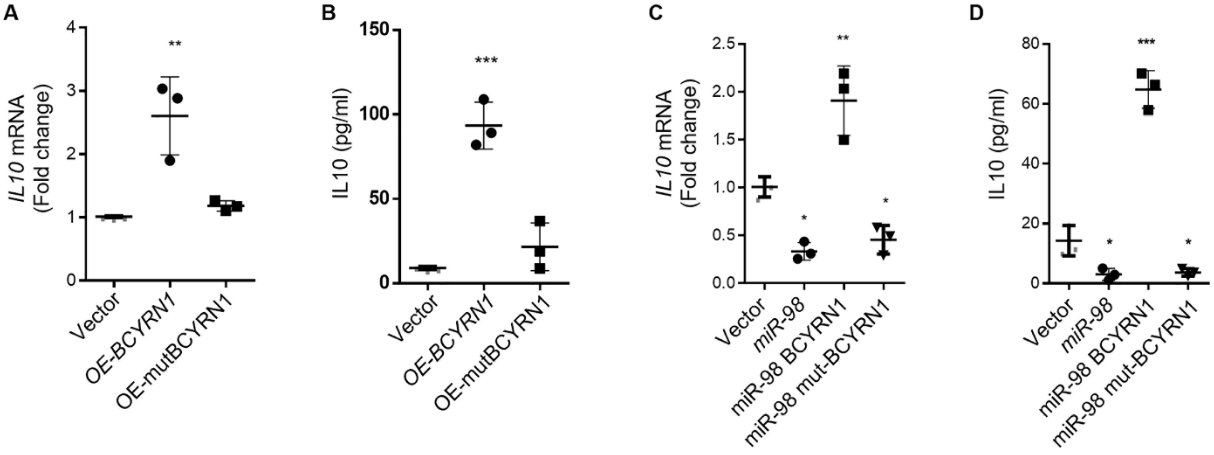

Figure S2 BCYRN1 construct with a disrupted miR-98 binding site (mut-BCYRN1) no longer suppresses IL10 levels mediated by miR-98.

(A, B) Human iTreg cells were transfected with Vector, OE-BCYRN1 lenti-vector, or OE-mut-BCYRN1 lenti-vector, followed by assessment of IL10 by qPCR (A) and ELISA (B). (C, D) Co-transfected vector, OE-BCYRN1 lenti-vector, or OE-mut-BCYRN1 lenti-vector with mimic miR-98 into human iTreg cells, followed by assessment of IL10 by qPCR (A) and ELISA (B). One-way ANOVA followed by Bonferroni's post hoc test was used to determine the statistical significance among multiple groups. *, $P < 0.05$, **, $P < 0.01$, ***, $P < 0.001$ versus vector alone group.

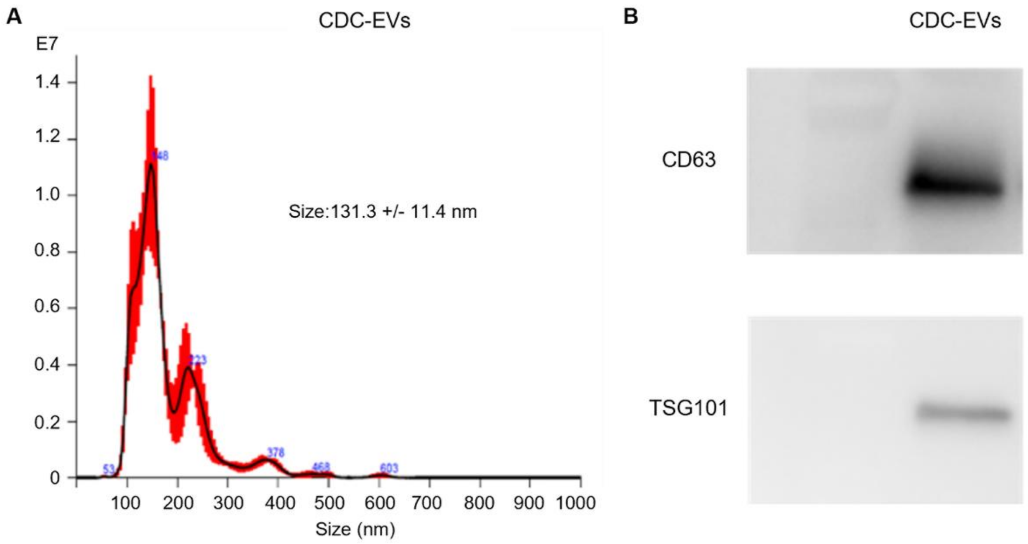

Figure S3 Characterization of CDC-EVs.

(A) The particle size distribution of CDC-EVs, as determined by nanoparticle tracking analysis (NTA). (B) Exosome markers CD63 and TSG101 detected by Western blot.

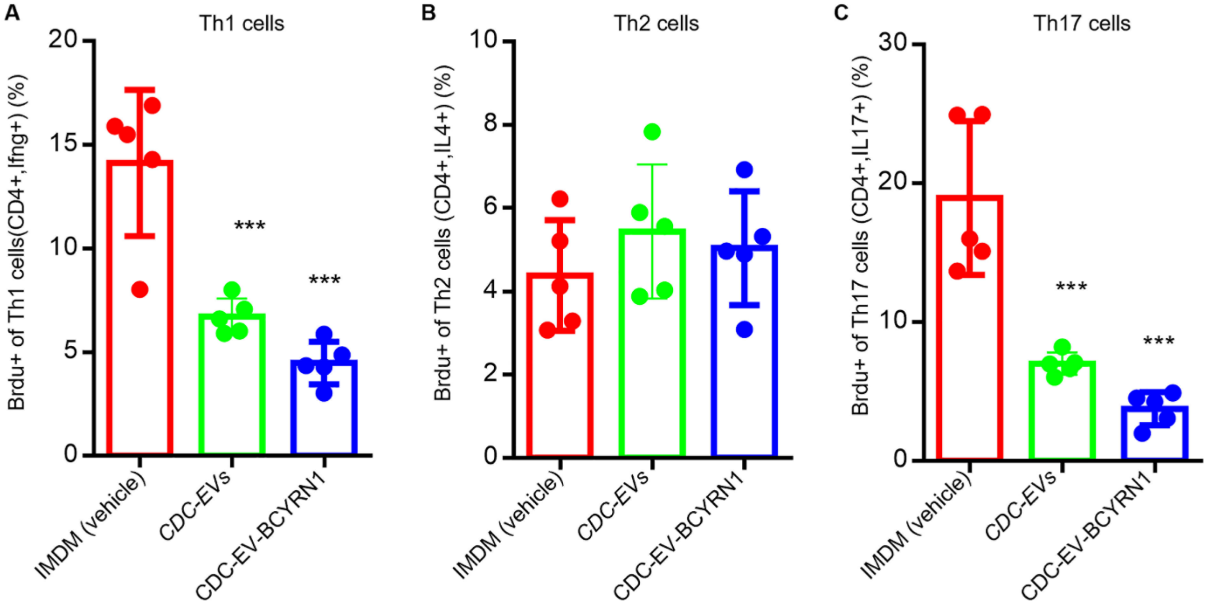

Figure S4 Enhanced expansion of Tregs could potentially contribute to the suppression of Th1 and Th17 proliferation *in vivo.*

(A-C) Pooled data of the CD4+IFNg+Brdu+ (A), CD4+IL4+Brdu+ (B), and CD4+IL17+Brdu+ populations in the heart from CDC-EVs, CDC-EVs overexpressing BCYRN1 (CDC-EVs-BCYRN1)- and IMDM (vehicle)-infused animals (n = 5 mice per group). One-way ANOVA followed by Bonferroni's post hoc test was used to determine the statistical significance among multiple groups. *, P < 0.05, **, P < 0.01, ***, P < 0.001 versus IMDM (vehicle) group.

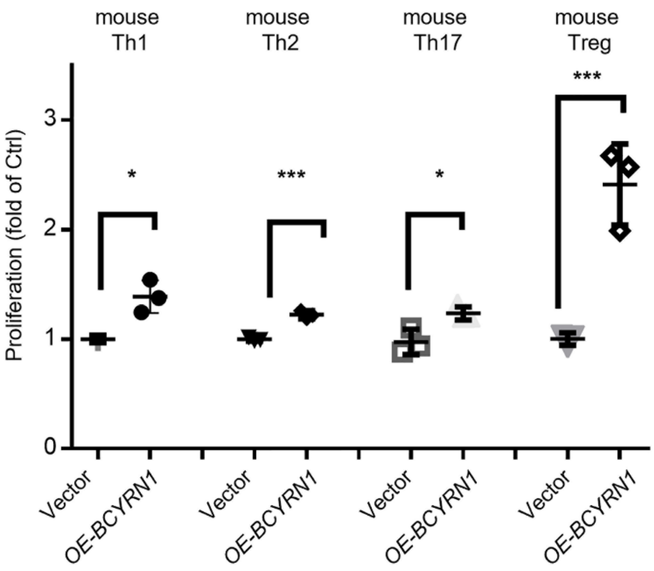

Figure S5 BCYRN1 overexpression increases the proliferation of Th1, Th2, Th17, and Treg cells.

Mouse iTreg cells were transfected with Vector, and OE-BCYRN1 lenti-vector, followed by assessment of proliferation using CCK assay. A two-tailed Student's t test was used to determine statistical significance in pairs of indicated groups. *, $P < 0.05$, **, $P < 0.01$, ***, $P < 0.001$ versus indicated vector group.
