# Supplementary figures and images for: Long noncoding RNA BCYRN1 promotes cardioprotection by enhancing human and murine regulatory T cell dynamics
Source: J Clin Invest. 2025 Mar 25;135(9):e179262. doi: 10.1172/JCI179262 (PMC12043100; doi:10.1172/JCI179262)

Fig. 4A

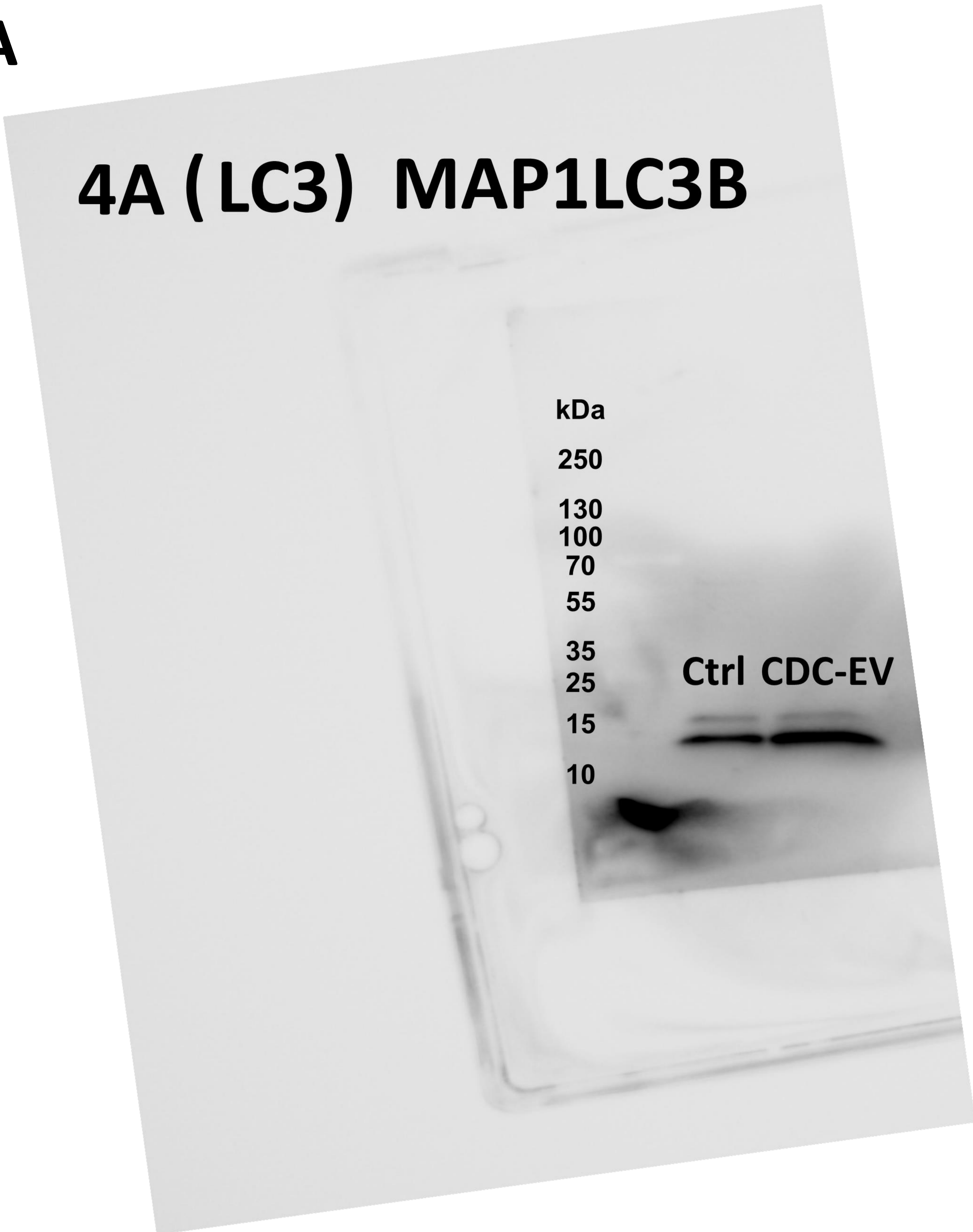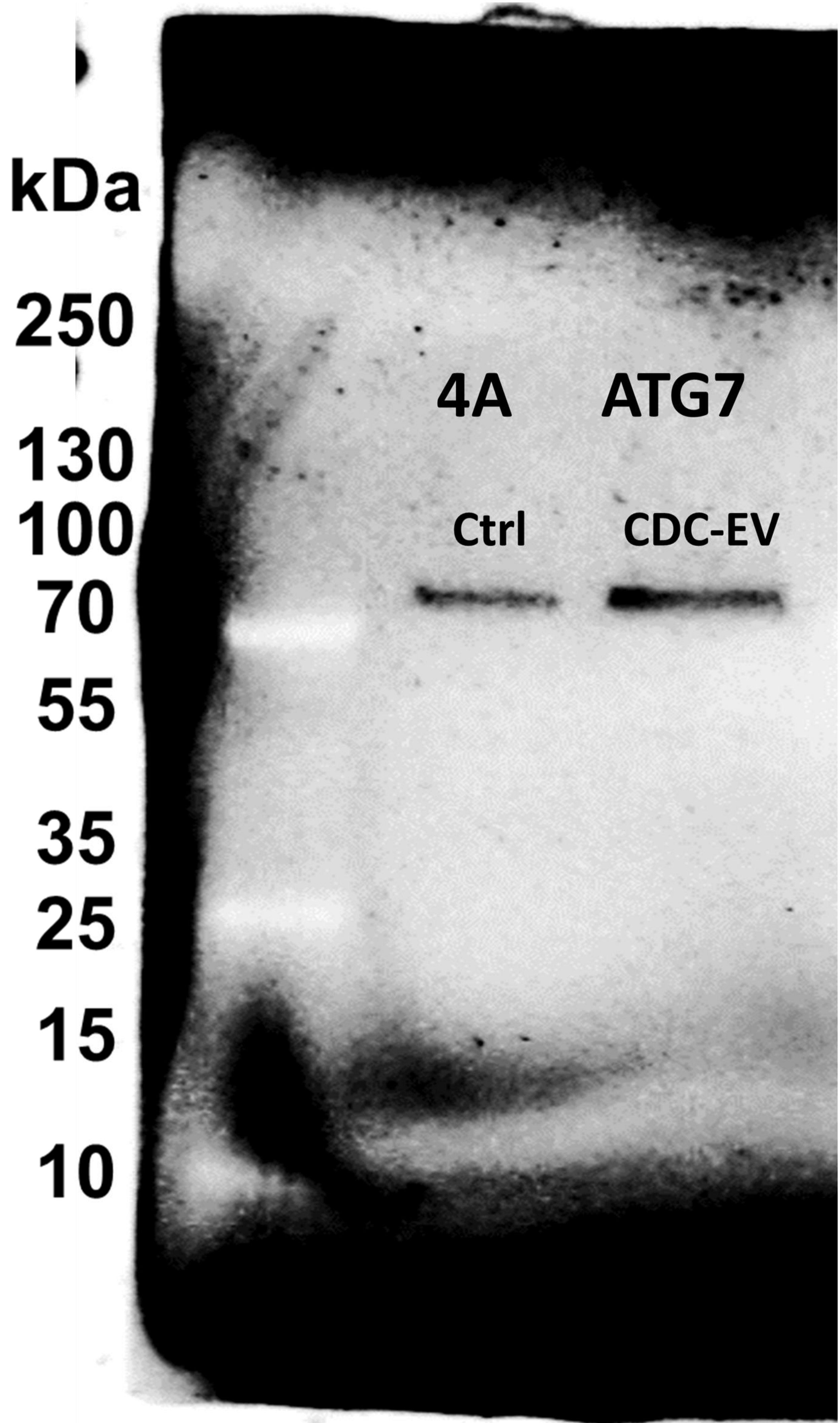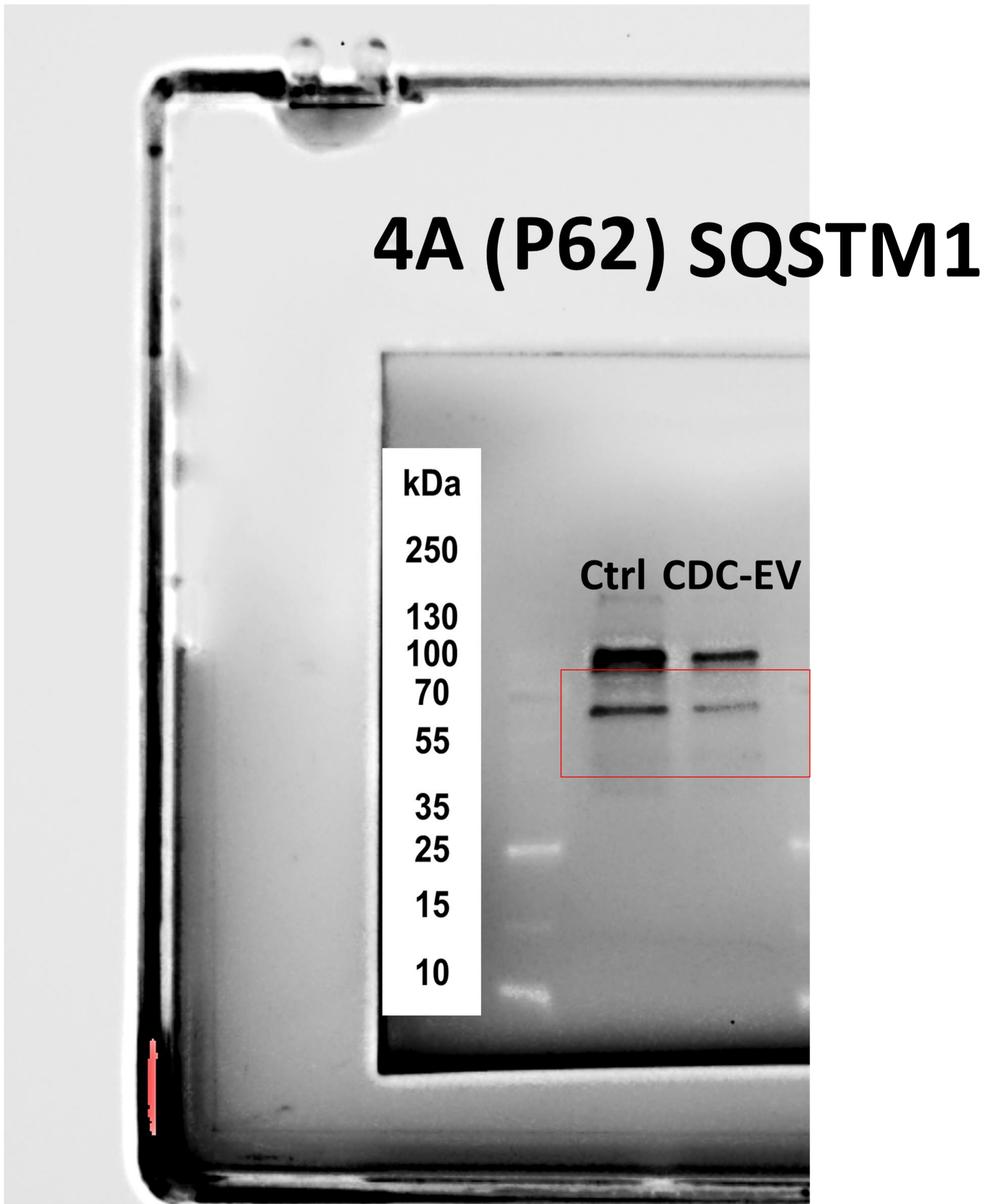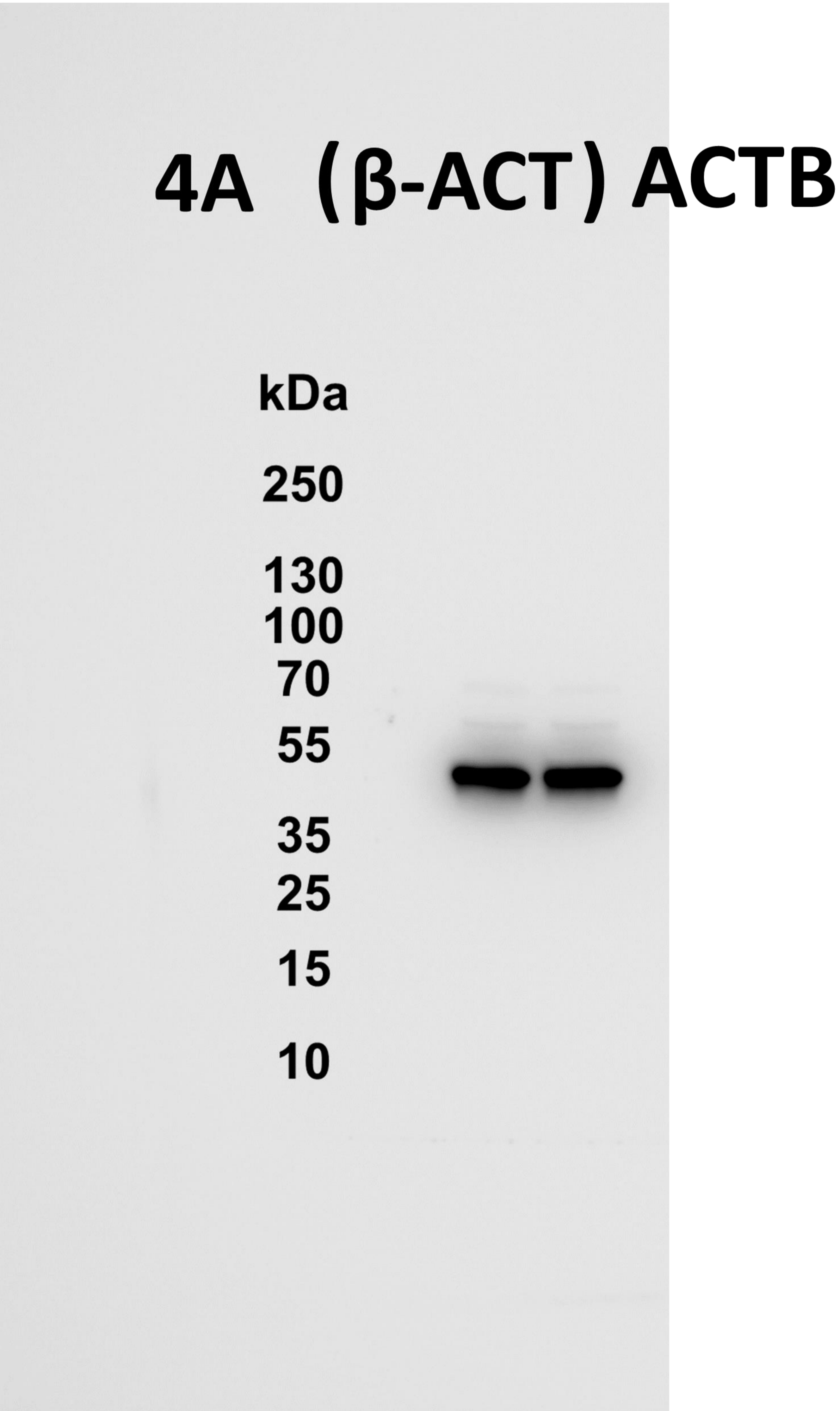

Fig. 4B

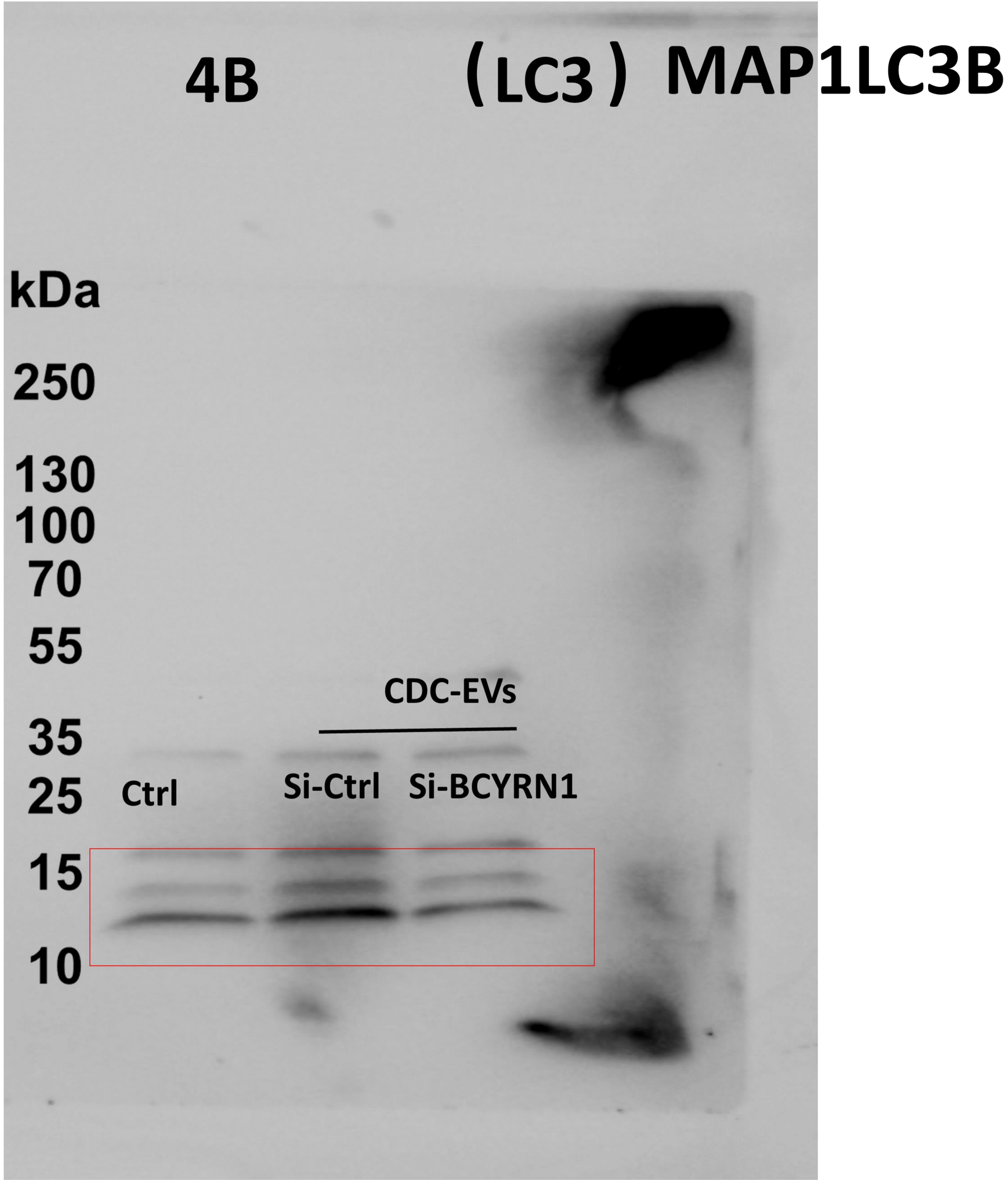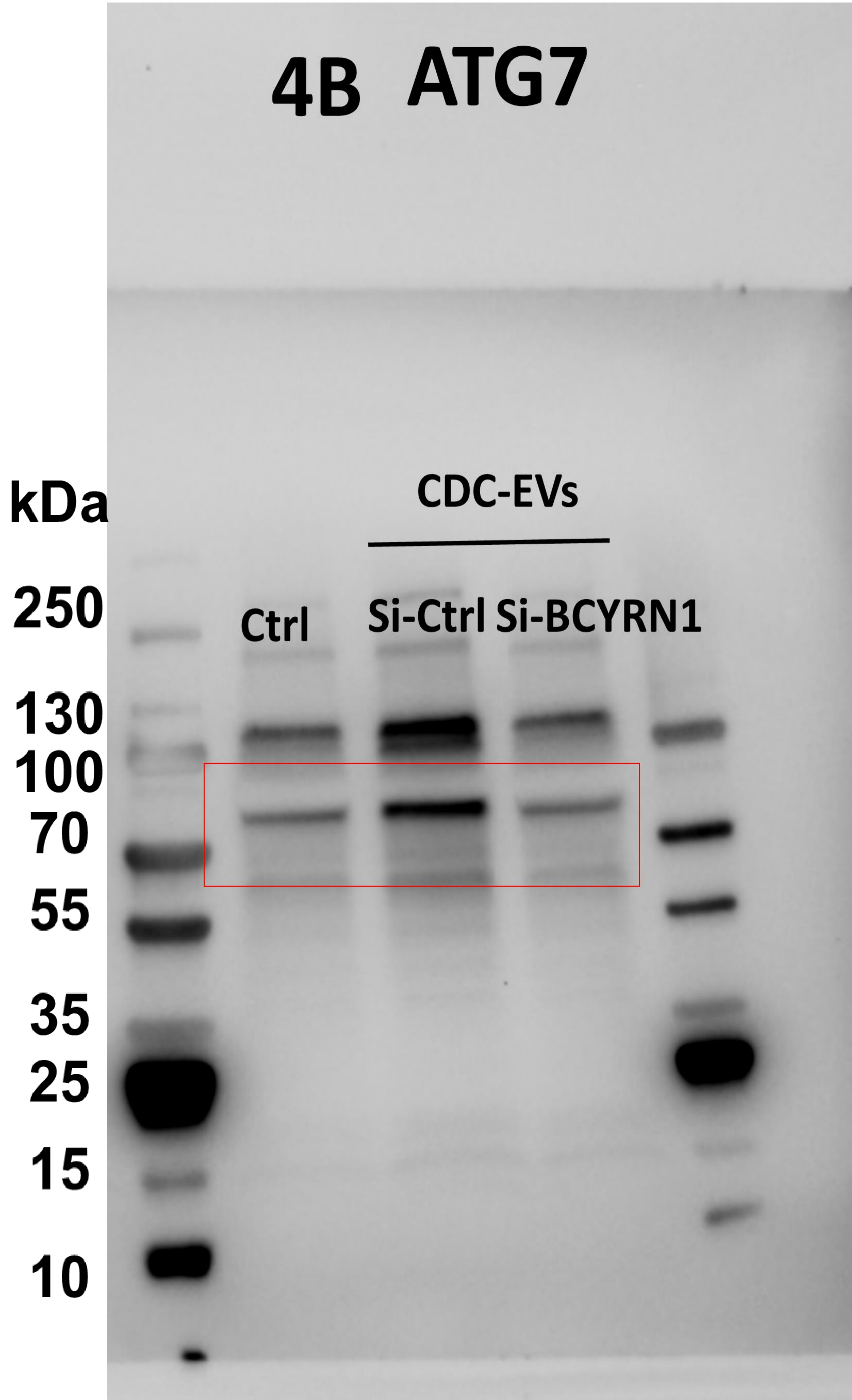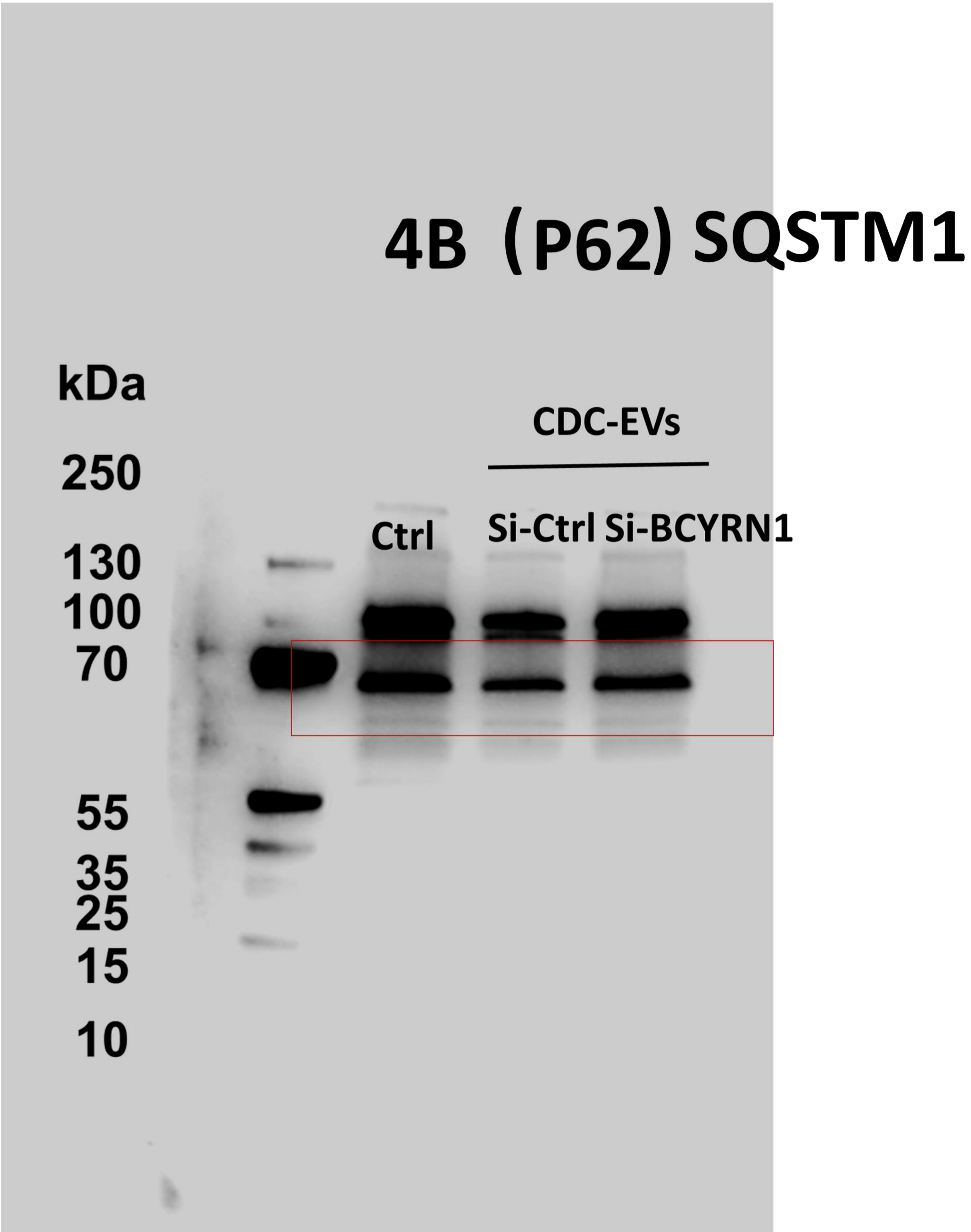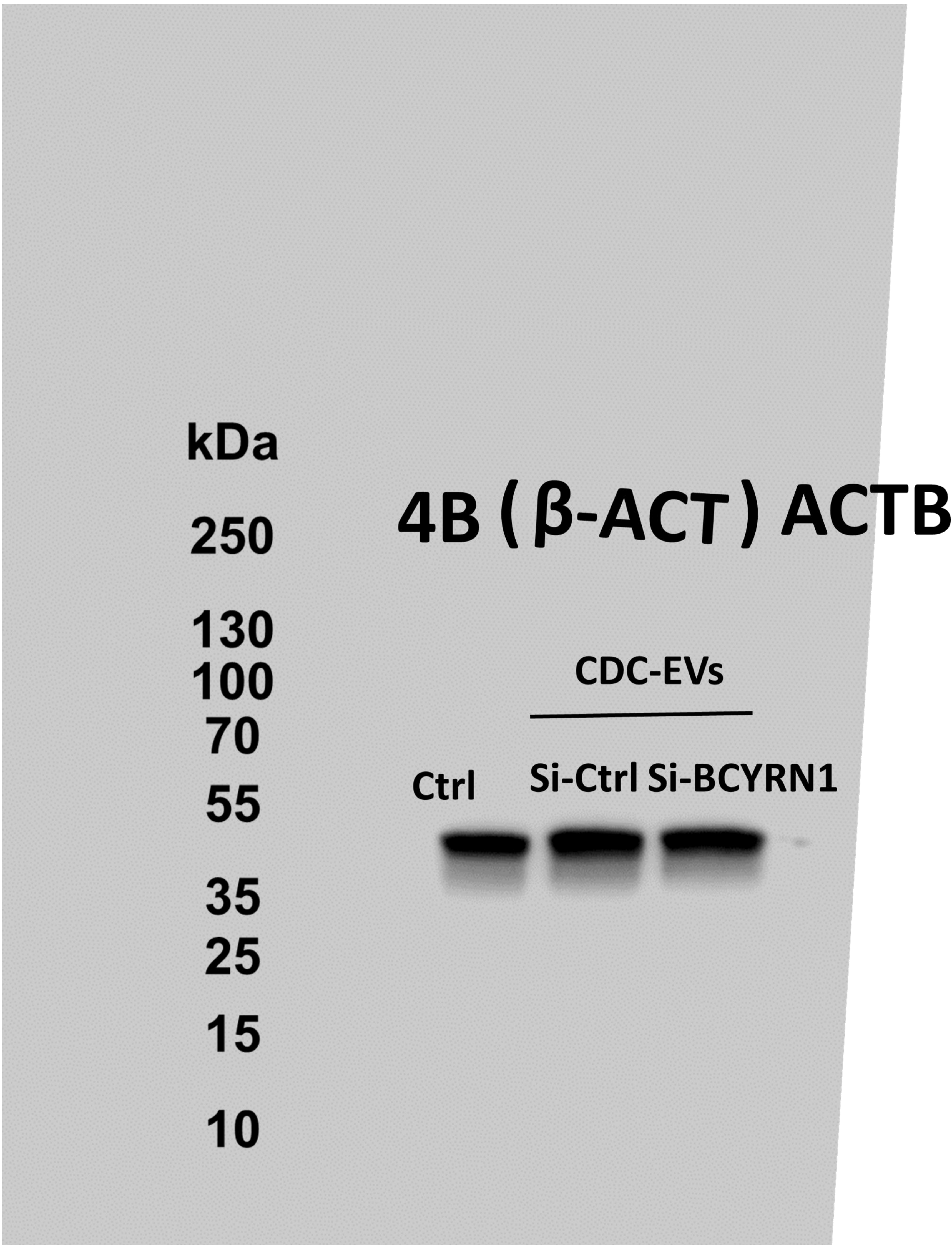

**Fig. 4C**

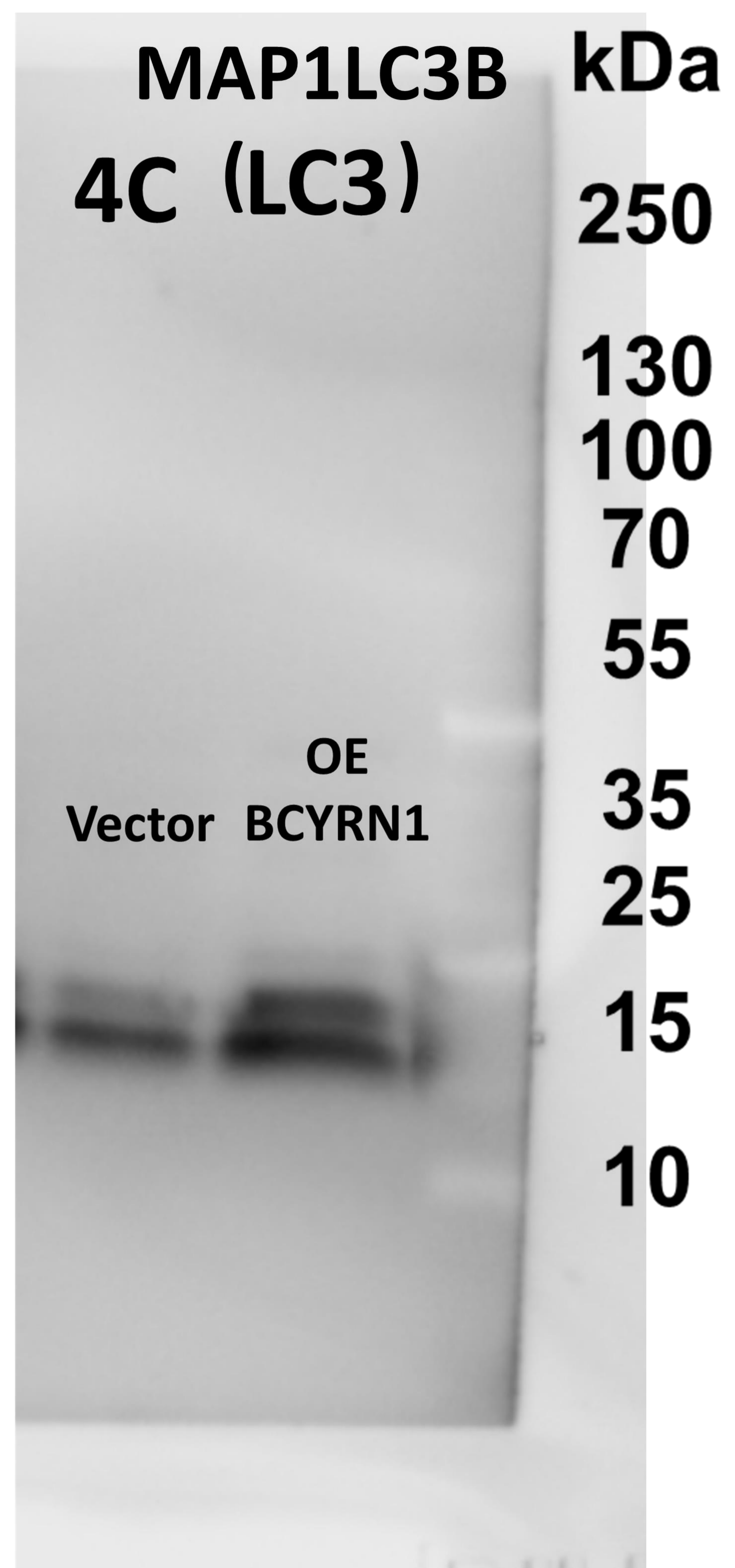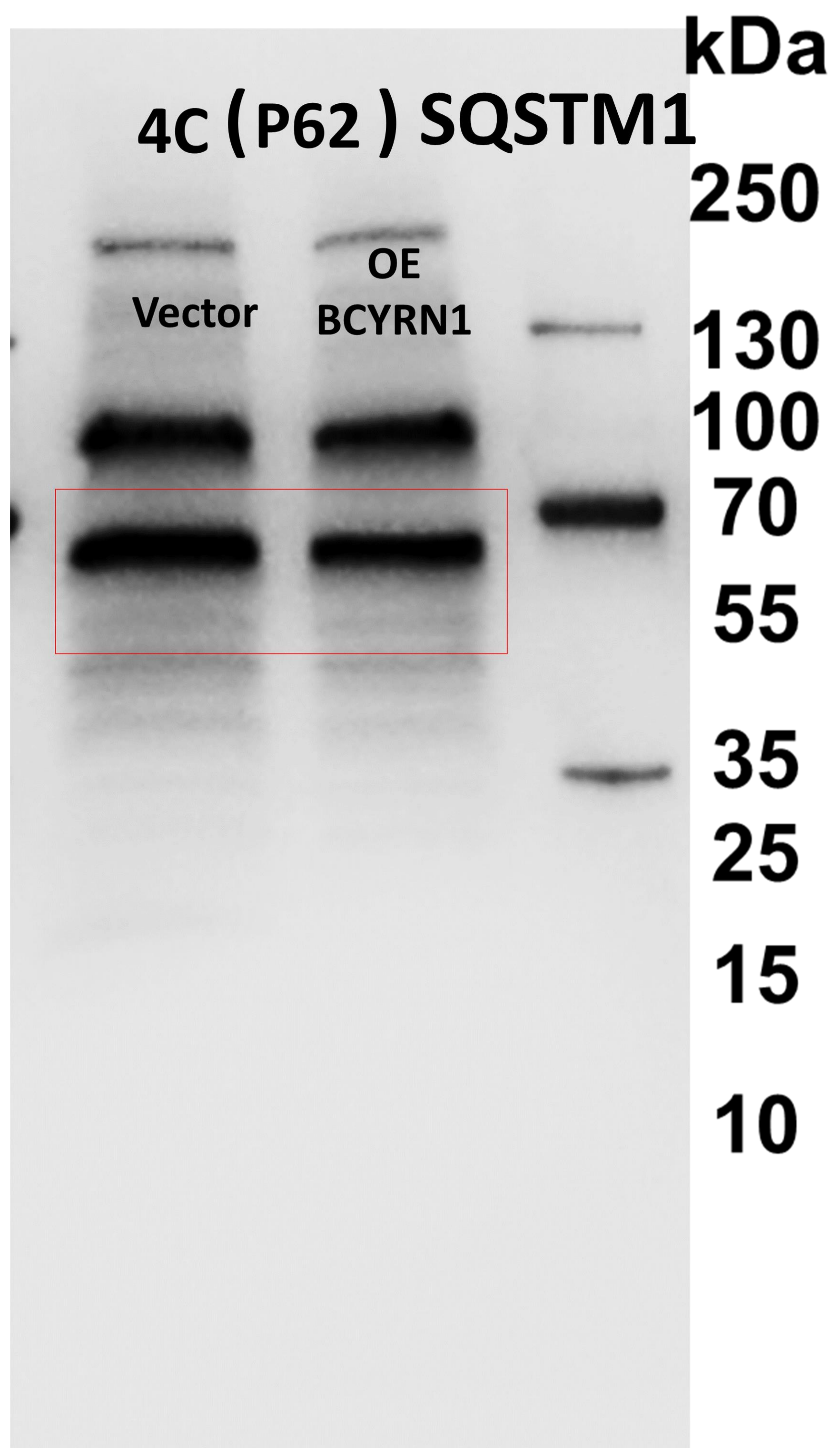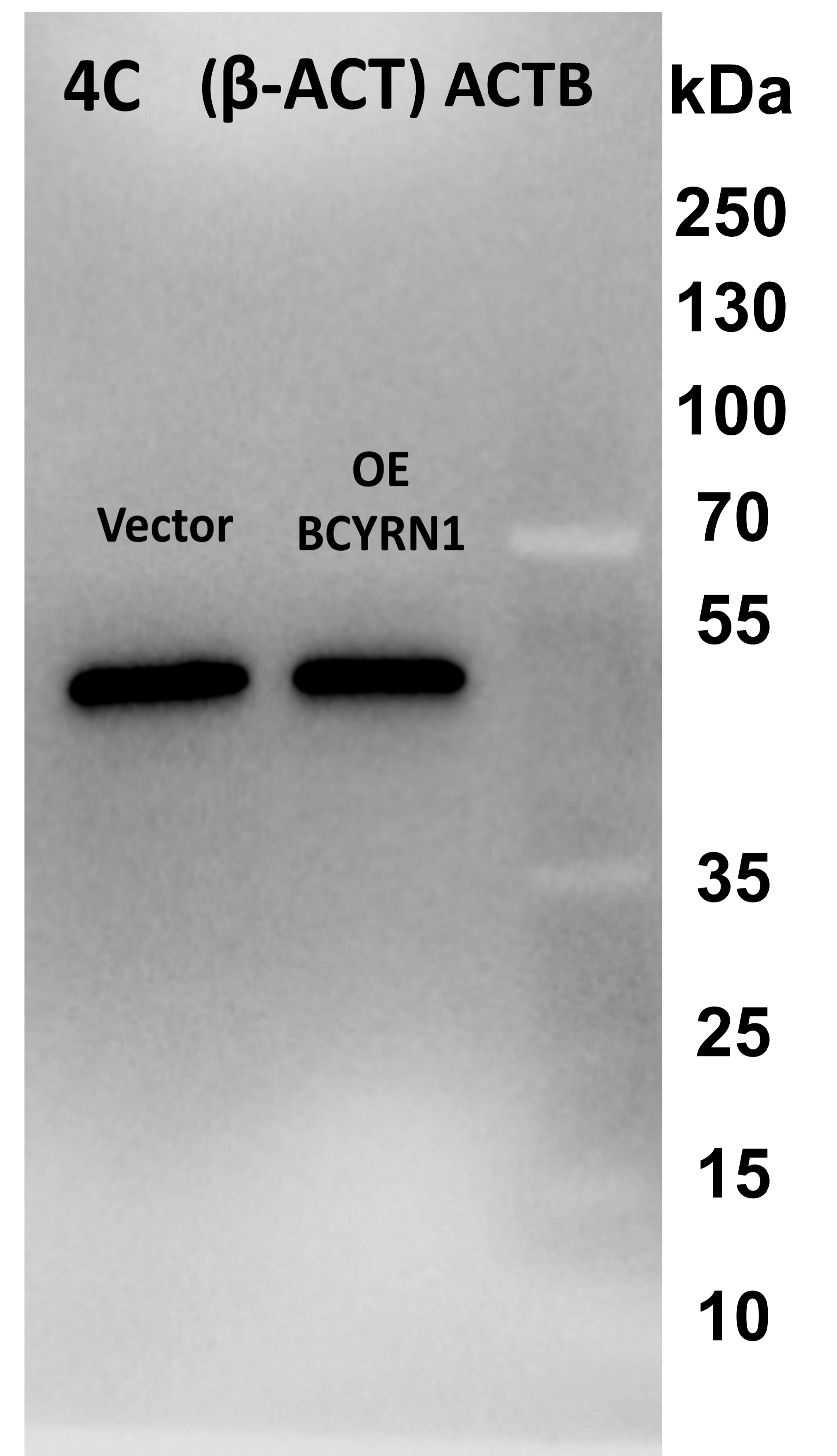

**Fig.4C     ATG7**

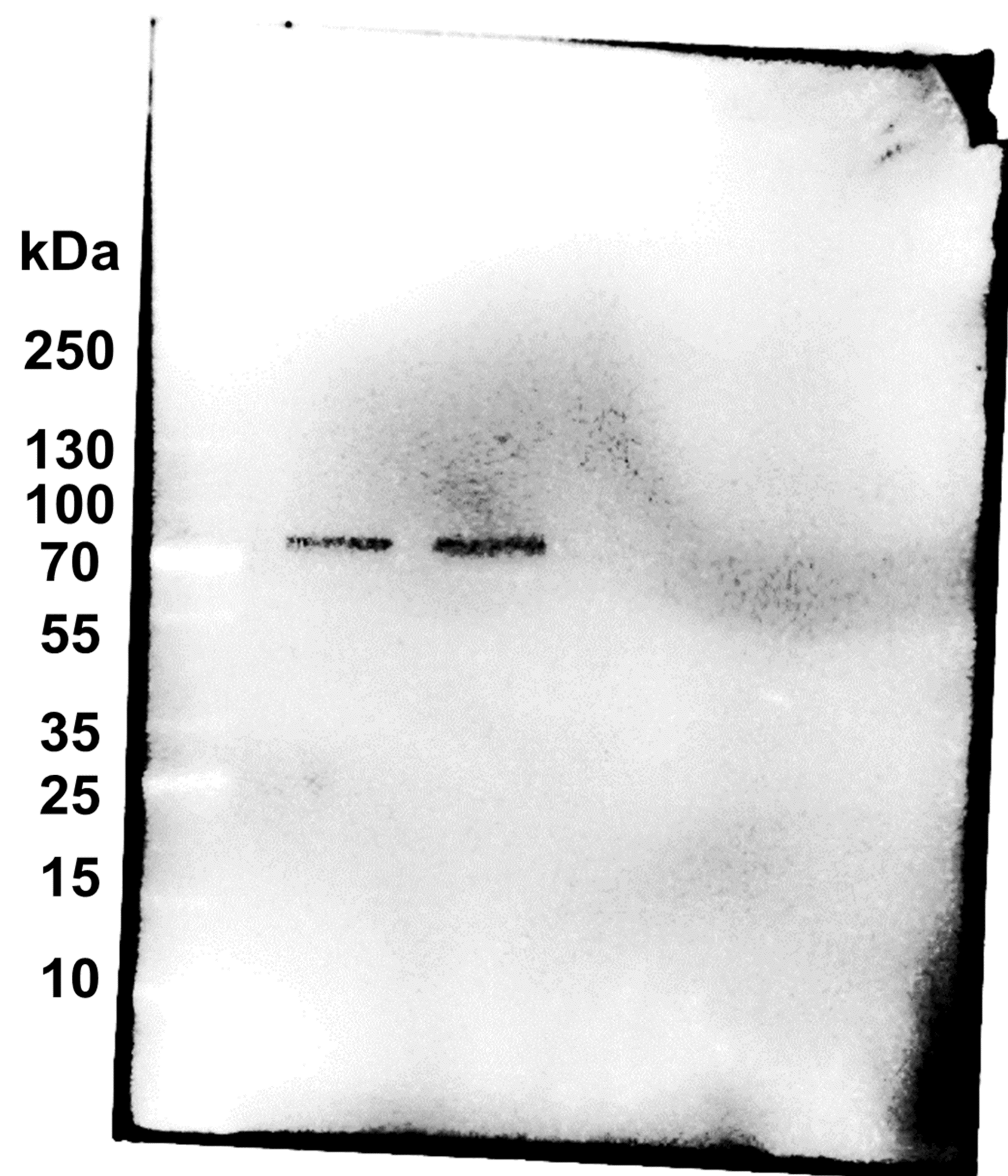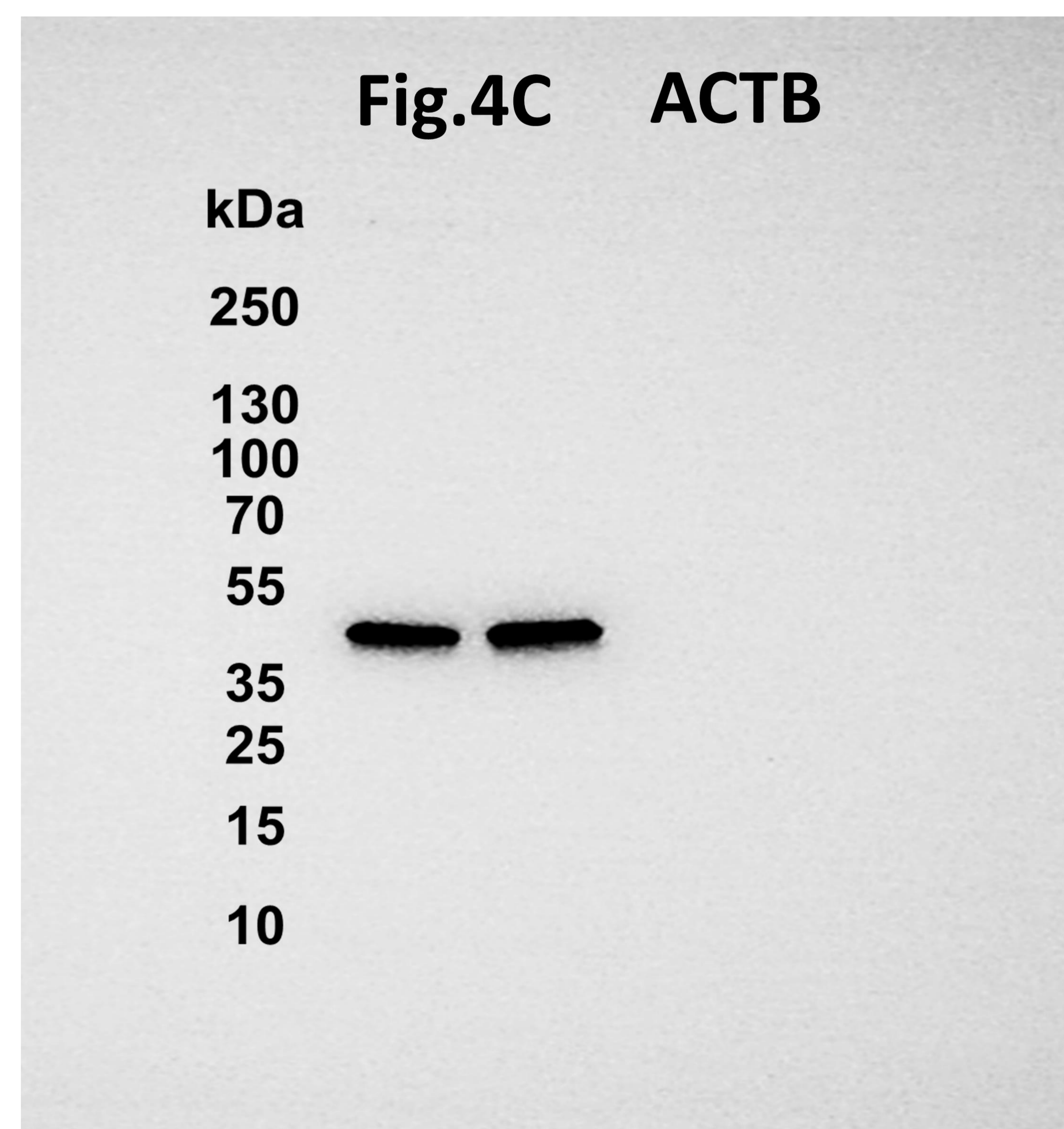

Fig. 4F

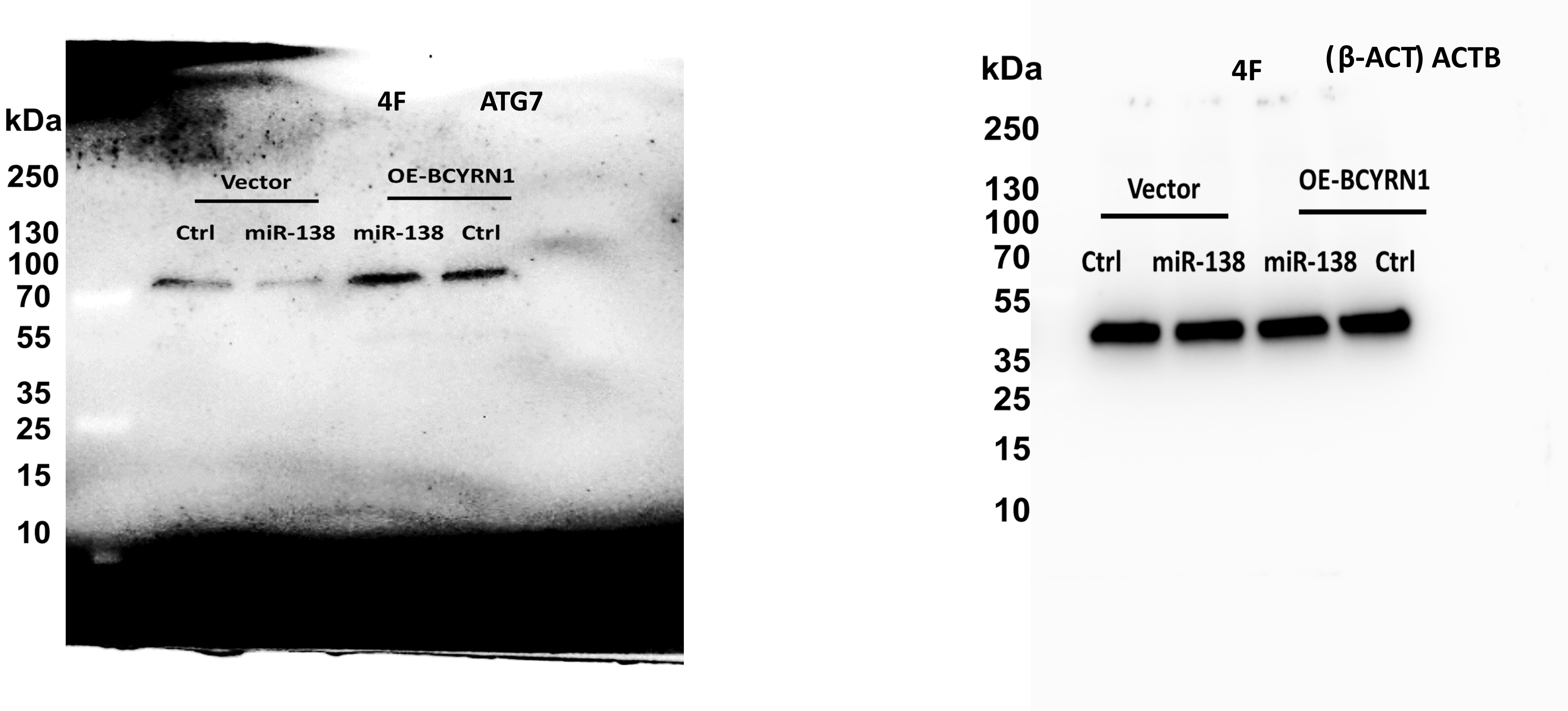

Fig. 5F

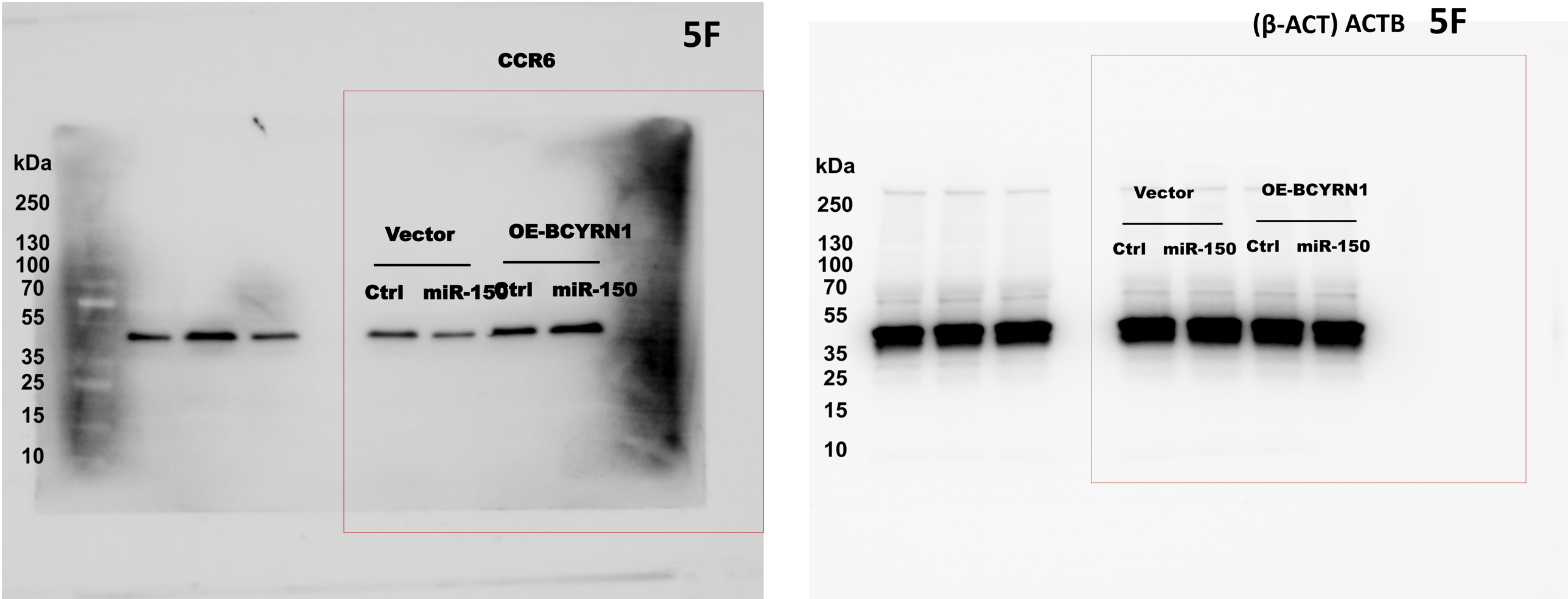

Supplement: Unedited blot and gel images [file jci-135-179262-s009.pdf]
